# Comparison of Equilibrium and Nonequilibrium Approaches for Relative Binding Free Energy Predictions

**DOI:** 10.1021/acs.jctc.3c00842

**Published:** 2023-10-20

**Authors:** Shunzhou Wan, Agastya P. Bhati, Peter V. Coveney

**Affiliations:** † Centre for Computational Science, Department of Chemistry, University College London, London WC1H 0AJ, U.K.; ‡ Advanced Research Computing Centre, University College London, London WC1H 0AJ, U.K.; § Computational Science Laboratory, Institute for Informatics, Faculty of Science, University of Amsterdam, Amsterdam 1012 WP, Netherlands

## Abstract

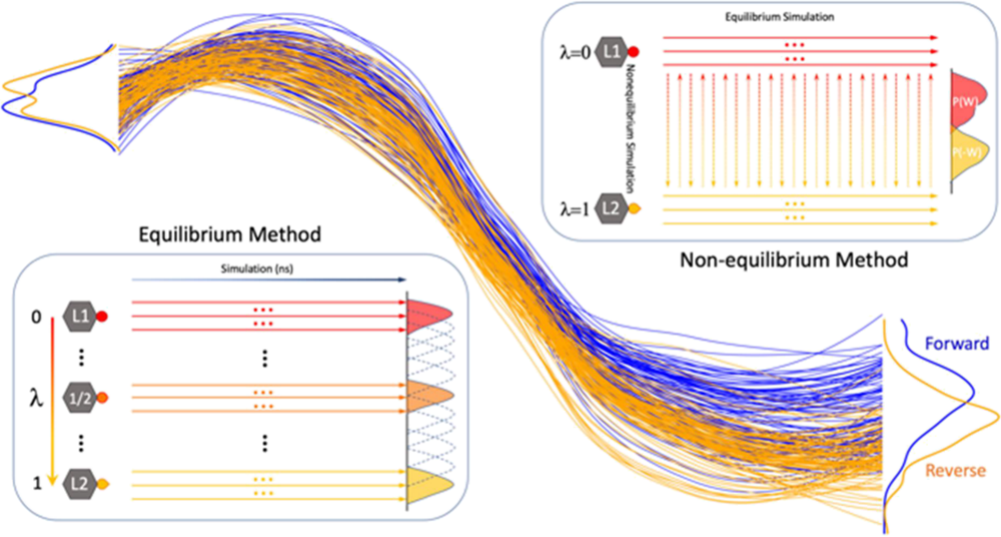

Alchemical relative binding free energy calculations have recently found important applications in drug optimization. A series of congeneric compounds are generated from a preidentified lead compound, and their relative binding affinities to a protein are assessed in order to optimize candidate drugs. While methods based on equilibrium thermodynamics have been extensively studied, an approach based on nonequilibrium methods has recently been reported together with claims of its superiority. However, these claims pay insufficient attention to the basis and reliability of both methods. Here we report a comparative study of the two approaches across a large data set, comprising more than 500 ligand transformations spanning in excess of 300 ligands binding to a set of 14 diverse protein targets. Ensemble methods are essential to quantify the uncertainty in these calculations, not only for the reasons already established in the equilibrium approach but also to ensure that the nonequilibrium calculations reside within their domain of validity. If and only if ensemble methods are applied, we find that the nonequilibrium method can achieve accuracy and precision comparable to those of the equilibrium approach. Compared to the equilibrium method, the nonequilibrium approach can reduce computational costs but introduces higher computational complexity and longer wall clock times. There are, however, cases where the standard length of a nonequilibrium transition is not sufficient, necessitating a complete rerun of the entire set of transitions. This significantly increases the computational cost and proves to be highly inconvenient during large-scale applications. Our findings provide a key set of recommendations that should be adopted for the reliable implementation of nonequilibrium approaches to relative binding free energy calculations in ligand-protein systems.

## Introduction

Free energy calculations are an essential tool by means of which to evaluate the binding affinity of drugs to their target proteins, and their applications are becoming increasingly widespread in the pharmaceutical industry as well as in clinical settings.^1−3^ This approach is by now well known, at least in principle. Unfortunately, aside from the case of very few research groups, insufficient attention has been paid to the reliability, reproducibility, and uncertainty quantification of the methods used. In the past few years, ensemble approaches have been found to be successful in generating reproducible and reliable predictions in conjunction with statistically robust uncertainty quantification.^4−6^ We have shown recently that the ensemble-based alchemical approach TIES (thermodynamic integration with enhanced sampling)^7^ generates relative binding free energies with chemical accuracy in all cases for a large demonstration data set.^8^ Such ensemble approaches are the only reliable way to compute statistically robust relative binding free energies and are equally applicable to other free energy methods. Ensemble-based free energy perturbation (FEP), for example, produces free energy predictions as reliably as TIES does.^9^ An essential corollary is that we cannot rely on approaches based on one-off simulations despite the fact that many authors continue to report them. Enhanced sampling methods, such as REST2 (replica exchange with solute scaling) as implemented in the free energy perturbation approach FEP+ by Schrödinger Inc., have been shown to be unreliable.^10−12^ While the oft-vaunted FEP+ method continues to be reported in the context of one-off simulations and, indeed, one still reads many papers in which one or a small number of “repeats” are performed,^13^ a growing number of authors now recognize that ensembles are, in fact, essential.^5,14^

**Figure 1:**
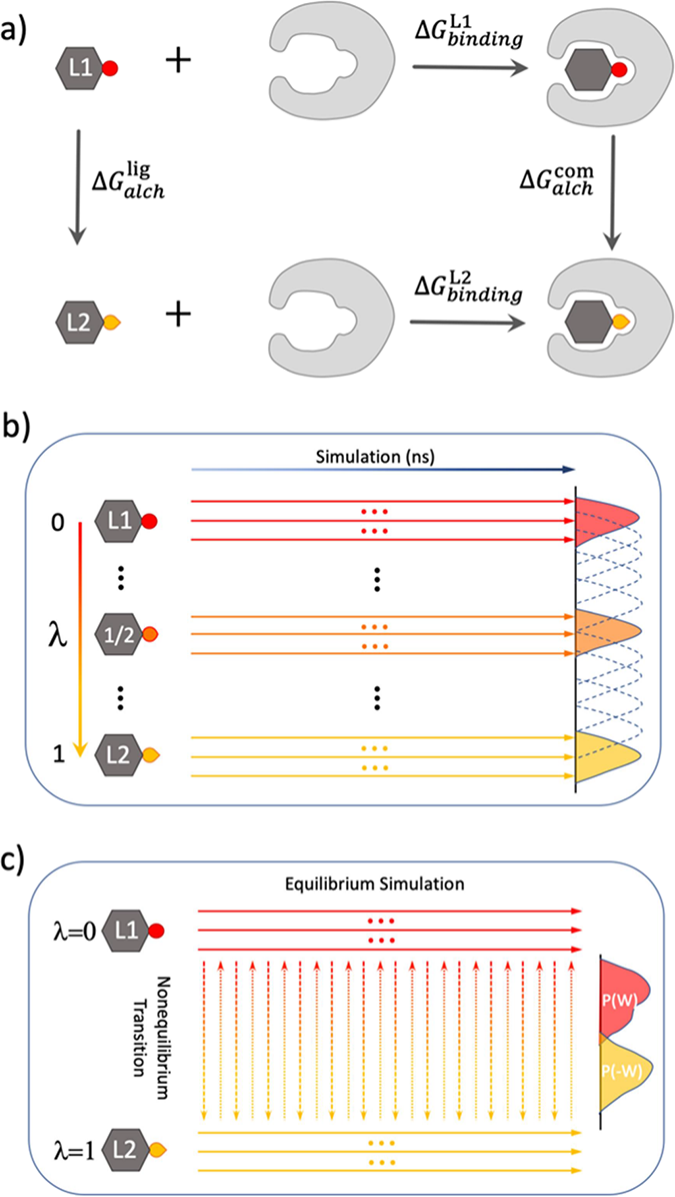
Free energy calculations. (a) Thermodynamic cycle for the calculation of relative binding free energy (ΔΔ*G*_bind_). The horizontal arrows correspond to the binding free energy of two ligands, L1 and L2, to the binding site of a protein. The vertical arrows correspond to the alchemical transitions of L1 to L2 in bulk solvent (solvent leg) and in the bound state (complex leg). The free energy changes in the alchemical process can be calculated using (b) an equilibrium or (c) a nonequilibrium method. In (b), intermediate states are introduced with 0 < λ < 1. Ensemble simulations are performed for each intermediate state, with individual simulations represented by an arrow symbol. In (c), two ensemble equilibrium simulations are run at the end point states (λ = 0 and λ = 1). Fast alchemical transitions are then performed to switch between the two end point states.

Alchemical free energy methods make use of a thermodynamic cycle (Figure 1a) that involves transforming one molecule into another through a series of intermediate alchemical states. The alchemical process can be performed using equilibrium or nonequilibrium methods (Figure 1b,c), from which the free energy difference between the initial and final states can be calculated. Equilibrium methods rely on the assumption that the system is at equilibrium for all intermediate states linking the two end point states. The free energy change associated with the gradual transformation of one state into another can then be calculated using methods such as FEP^15^ and thermodynamic integration (TI).^16^ Nonequilibrium methods carry out fast transitions driving the system irreversibly from one state into another and *versa vice*. The amount of work needed for these rapid alchemical transformations can be used to derive the free energy difference between the initial and final states using estimators based on Jarzynski’s equality,^17^ Crooks’ fluctuation theorem,^18^ and Bennett’s acceptance ratio (BAR)^19^ (which exploits Crooks’ nonequilibrium work theorem).

To carry out nonequilibrium free energy calculations, one first needs to perform independent equilibrium simulations at the two end point states. The end-state conformations need to be well sampled, as the accuracy of the free energy prediction depends on the convergence of the equilibrium sampling. While many studies in the literature use what are termed “long” simulations for this step, we recommend the use of ensemble simulations as we have established repeatedly that it is much more efficient for the sampling of conformational space than a single long simulation.^6^ Indeed, our recent study on long-time-scale simulations has shown that individual trajectories, with temporal durations up to 10 μs, do not allow for accurate or reproducible elucidation of macroscopic expectation values.^20^ In addition, using supercomputers, the ensemble members can all be executed concurrently in the wall clock time of a single short run. From the trajectories of equilibrium simulations at each end state, snapshots are extracted to initiate fast alchemical transitions in order to drive the system to the other end state. Such fast transitions force the system out of equilibrium and hence are termed “nonequilibrium transitions” hereafter. These nonequilibrium transitions are executed in both directions (λ: 0→1 and 1→0), and the work required to perform them is obtained to determine the free energy difference between the two end states (Figure 1c). The nonequilibrium method is significantly more complicated to implement than the equilibrium method because there are more “moving parts” in the approach (that is, there are additional settings and accompanying parameters to select). Indeed, one could be forgiven for asking why one should use nonequilibrium methods to compute an equilibrium quantity such as free energy. Clearly, it can only be justified if it proves easier and cheaper to implement and/or generates results that are more accurate and precise.

The nonequilibrium approach has been used as a general methodology for large-scale conformational sampling, usually with biasing forces added to the equations of motion. The approach has been recognized as a powerful tool for sampling rare events and providing fruitful insights into both equilibrium and nonequilibrium processes. Such rare events involve high-energy barriers or long-time-scale phenomena, making sampling challenging with equilibrium methods. In a nonequilibrium simulation, a system is driven away from thermodynamic equilibrium by a perturbation—a deviation arising from an external environment—which boosts a physical process of interest. Nonequilibrium ensemble methods have been used to identify the changing forces from the bound to the partially dissociated state of ligands from their target G protein-coupled receptor and to predict ligand-protein relative residence times.^21^ We ourselves won the first HPC Analytics Challenge Award at the Supercomputing Conference 2005 for our study of free energy estimation for the translocation of polynucleotides through α-hemolysin nanopores.^22,23^ The work received recognition precisely because it sought to take advantage of such nonequilibrium methods in order to parallelize the calculation of a free energy profile along the pore. Had we sought to determine this using a single equilibrium simulation, the runs might still be executing now. It was one of the first times that we used replicas and ensembles to determine free energies.

In the past few years, nonequilibrium methods have been increasingly used in alchemical simulations, where intermediate alchemical states are introduced to bridge high-probability regions of configuration space (Figure 1c). The free energy differences between the two regions can then be calculated. The approach can be used for a relative binding free energy (RBFE) between two compounds to the same target protein when the two regions represent the bound states for the two compounds; it can also be used for an absolute binding free energy (ABFE), where one region represents a bound state while another is an apo state of the protein.

Although many studies assert that the nonequilibrium approach attains comparable levels of accuracy and convergence to the equilibrium method, it is also often touted for its claimed advantage in terms of computational efficiency.^24^ However, the gain in computational efficiency depends on the computational protocols used in such studies, including the duration of the switching process^25,26^ and the switching method applied^27^ (including the use of different soft-core potentials^9^). Unfortunately, a rather bewildering set of differing if not wholly inconsistent recommendations has been provided for nonequilibrium simulations.^13,28,29^ For the number of snapshots to be extracted from the equilibrium trajectories at each end state to initiate nonequilibrium alchemical transitions, it has been suggested that 50–100 snapshots are generally sufficient for RBFE calculations;^28^ yet one and the same group has variously used 150 and 80 snapshots.^13,29^ While authors frequently mention that caution is needed concerning the possibility of insufficient sampling due to short simulations, most of these nonequilibrium transitions simply invoke short simulation times (usually less than 100 ps) on grounds of expediency alone. While several authors have begun to recognize the necessity of using ensembles, the number of replicas in such ensembles has not been satisfactorily addressed. Indeed, the term “ensemble” is not used in many cases. Although ensemble methods are increasingly being recognized as the only sure-fire way of reporting reproducible results in the context of molecular dynamics, it is common to see the word “repeats” (as used experimentally) instead of ensembles or replicas in published papers. In the literature, various different ensemble sizes have been used, from one (i.e., “one-off” simulations) to 3^13^ or 5.^29^ The primary motivation for these choices is based on minimizing the associated computational cost rather than on the accuracy and reliability of the ensuing predictions. Moreover, it has been reported^30^ that short transition times lead to a heavily biased estimate for predictions of binding free energy changes in protein–protein complexes. These predictions do not converge until the nonequilibrium simulation time reaches 5 to 8 ns for the two complexes studied. Compounding this, the equilibrium simulations at the end points are relatively long (40 ns), and they are performed as one-off simulations,^30^ an approach commonly used in many similar studies. As we have repeatedly explained over the past several years, the need for ensembles is intrinsic to molecular dynamics methods since the dynamics is chaotic and mixing in the hierarchy of ergodic theory: trajectories and quantities of interest extracted from these display extreme sensitivity to the initial conditions.^4,6,20^ This applies irrespective of the duration of the simulation under investigation^20^ and renders one-off simulations not reproducible.

The purpose of the present paper is to assess the performance of nonequilibrium alchemical methods for relative free energy predictions and to examine the impact of simulation parameter settings on their predictions. Here we use the reference data set from previous publications^8^ to look at the predictions from the nonequilibrium method and compare them with prior results obtained from equilibrium calculations. The paper is structured as follows: in the next section, we lay out the methods used, while in the following one, we present the results and our recommendations for the nonequilibrium simulation settings. The article ends with our conclusions from the study.

## Methods

### Data Set

We evaluate the nonequilibrium method described above by applying it to the aforementioned data set, comprising 503 ligand pairs bound to a diverse set of 14 pharmaceutically relevant protein targets (Table S1). The ligand perturbations include a wide range of chemical modifications typically seen in medicinal chemistry research. The set of proteins and compounds has become a benchmark for free energy predictions, being used recently, wholly or partially, in large-scale RBFE studies using TIES,^8^ the original and subsequent FEP+ studies,^31,32^ and the comparison of equilibrium and nonequilibrium simulations with FEP+ and pmx (a python library used to set up and analyze MD simulations with GROMACS),^13^ and in alchemical ABFE studies.^24,33^

### Nonequilibrium Approach

We adapt TIES,^7,34^ the equilibrium method we have developed, to perform nonequilibrium simulations for the calculation of binding free energy changes corresponding to an alchemical transformation. A thermodynamic cycle approach (Figure 1a) is invoked for both the equilibrium and nonequilibrium methods, in which the compound pair is transferred from one to another alchemically, both in an aqueous solvent and when bound to a protein. The physical bound states are linked through a series of nonphysical intermediate states; this entire alchemical transformation is controlled using a parameter, λ, such that λ = 0 and 1 denote the two physical end states, whereas 0 < λ < 1 correspond to the intermediate states. For the equilibrium TIES approach (Figure 1b), equilibrium simulations are performed at each intermediate state. We have established a standard protocol in which an ensemble of 5 replicas is performed at each λ window.^7^ It should be noted that, in practice, this standard setting may need to be adjusted in some cases to control uncertainties and their computational cost.^35^ For the nonequilibrium approach (Figure 1c), fast transitions are performed to drive the system in the forward (λ 0→1) and reverse (λ 1→0) directions without requiring the system to reach equilibrium throughout the transition. The Crooks’ fluctuation theorem, Jarzynski’s equality, or BAR estimators can then be employed to obtain free energy differences between the two physical states. The physical end point simulations may be reused in cases when the equilibrium conformations are generated for a common end point state without a hybrid topology.^36^ It is, however, more common that the “physical” end point states in alchemical studies retain dummy atoms that are covalently attached to the system but have no nonbonded interactions with their environment. The dummy atoms are different for a given compound, L_0_, pairing with different compounds, L*_x_* (*x* = 1, 2, 3, ...). For these compound pairs L_0_–L_*x*_, the “physical” L_0_-end points are therefore different, and the equilibrium simulation of the L_0_-end point for one of the L_0_–L*_x_* pairs cannot be used for others directly.

While we use the standard TIES protocol^7^ for the equilibrium method, the choices of parameters in nonequilibrium simulations are informed by our extensive systematic analysis for a subset of the molecular systems (Table S2) which serves as an evaluation for the computational efficiency of the method together with the convergence and the accuracy of the predictions. We have systematically varied several key parameters specific to the nonequilibrium approach, including the ensemble size and simulation length of the equilibrium simulations at the end points, the duration of the nonequilibrium transition, and the number of snapshots used per ligand pair. For the entire set of molecular systems, we used 5 replicas for the ensemble equilibrium simulations at the physical bound states, followed by nonequilibrium transitions from one physical state to another (Figure 1c). The choice of the ensemble size is determined by the extensive analysis of the subset and also informed by extensive applications of TIES,^7−9,11,34,37−39^ which have demonstrated that its size ensures a robust sampling of the equilibrium state corresponding to the initial structure. The nonequilibrium transitions are initiated from snapshots extracted from the equilibrium trajectories at the two physical states. We have performed 10 ns production runs for each equilibrium replica at the two end points, from which snapshots are extracted to initiate nonequilibrium transitions of lengths varying from 50 ps to 2 ns in protein and solvent environments (Table S1). Nonequilibrium transitions are run in both forward and reverse directions using 100 snapshots each for a single ligand pair. The snapshots are extracted evenly from the equilibrium simulations at the end points.

Computational efficiency is frequently asserted to be one of the most important advantages of the nonequilibrium method, which is achieved by short simulation times for the fast transitions of one state into another. The equilibrium method with the TIES protocol uses 5 replicas, 13 λ-windows, and a 4 ns production run for each window, amounting to a total production simulation runtime of 260 ns^8^ (Table 1). Based on our extensive analysis and resulting recommendations for the various parameters, each free energy calculation using the nonequilibrium method requires a simulation time of 150 ns. However, there are cases where this proves insufficient, as longer nonequilibrium transitions are needed. When the duration of the transitions increases from 250 ps (generally recommended; see details in the Results section) to 2 ns, the total production run increases to 500 ns for each ligand pair. It should be noted that the computational efficiency for the equilibrium method can be enhanced by optimizing the λ distribution in the [0,1] interval, where the number and location of the λ windows may be determined adaptively.^40^ On a modern supercomputer, all production runs in the equilibrium method can be executed concurrently, i.e. the wall clock time required is that of a single 4 ns run, which is about 6–8 h using CPUs and <1 h using a single GPU for proteins of typical size (250–350 residues). For the nonequilibrium approach, however, the equilibrium simulations at the end points need to be completed before the nonequilibrium transitions can be executed. This means that the completion time for one nonequilibrium calculation is no less than a single 10.25 ns run (10 ns equilibrium followed by a 250 ps nonequilibrium run, assuming zero waiting time in between), which is no less than two and a half times the wall clock time of the equilibrium method (Table 1). It should be noted that, unlike the equilibrium approach, the nonequilibrium approach also has a severe practical limitation. If the conformational sampling is found insufficient, in the equilibrium method, more simulations can be readily added by inserting more intermediate λ-windows and/or extending simulations at selected λ-windows; in the nonequilibrium method, however, the entire sequence of alchemical transitions from one end point to the other needs to be rerun if their current duration is found insufficient. This is a major bottleneck in large-scale applications of the nonequilibrium method where a “one-size fits all” approach is not feasible and flexibility in the protocol is paramount.

**Table 1:** Computational Cost and Wall Clock Time for Simulations of Ligand-Protein Complex Using Equilibrium and Nonequilibrium Methods

method	protocol		total simulation	wall clock time^a^
equilibrium	5 replicas		260 ns	∼45 min
	13 λ-windows			
	4 ns production run for each window			
nonequilibrium	equilibrium runs:	nonequilibrium transition:	500 ns (150 ns)^b^	∼135 min(∼115 min)^b^
	2 end points	2 directions		
	5 replicas	100 runs each direction		
	10 ns per replica	2 ns (250 ps)^b^ each run		

a All individual runs are executed concurrently wherever possible. Wall clock time is shown as the execution time on ALCF’s Polaris, an HPE Apollo 6500 Gen10+ system equipped with NVIDIA A100 GPU accelerators.

b Simulation protocol recommended from current study (see Results section).

All of the molecules were prepared in our previous study^8^ where details can be found on the molecular systems and the equilibrium simulations. We provide a brief summary of these systems below. The general Amber force field 2 (GAFF2) was used for drug parametrizations with the assignment of AM1-BCC partial charges using the Antechamber component of the AmberTools package.^41^ The Amber ff14SB force field was used for the proteins and TIP3P for the water molecules. All systems were solvated in orthorhombic water boxes with a minimum extension from the protein of 14 Å. To avoid the well-known “end-point catastrophe”, a soft-core potential was used for pairwise van der Waals interactions involving the perturbed atoms. No soft-core potential was applied to the electrostatic interactions, which (de)couple at a faster pace than the vdW interactions so that the partial charges on perturbed atoms were removed before they were fully annihilated and the charges on the growing atoms were introduced after they appeared.^42^ For the current study, the majority of the simulations were performed with NAMD3 using one GPU for each individual MD simulation on Polaris at Argonne Leadership Computing Facility; some simulations were performed with NAMD2.14 using up to 96 CPUs per MD simulation on SuperMUC-NG at the Leibniz Supercomputing Centre. Langevin dynamics was used to maintain the temperature at 300 K with a friction coefficient of 5 fs^–1^. A Langevin piston was used as the barostat, with a piston period of 200 fs and a piston decay of 100 fs. A nonbonded cutoff of 12 Å was used with a switching distance of 10 Å. The PME algorithm was used to calculate the electrostatic contribution to the potential. A 2 fs time step was used for all of the MD simulations.

In the case of the equilibrium simulations, the systems were first minimized with all heavy protein atoms restrained at their initial positions and restraining force constants related to their β-factors in X-ray structures. A 2 ns equilibration run was performed within an NPT ensemble during which the restraints on heavy atoms were gradually removed. Finally, 10 ns production simulations were executed, with snapshots saved every 20 ps. One hundred snapshots were then extracted evenly from the ensemble of five replicas, from which nonequilibrium transitions with different simulation lengths were initiated. For the 2 ns nonequilibrium transitions, the alchemical parameter λ was changed by 0.002 every 4 ps, with 501 energy derivatives (including both end points) stored for further analyses. The variation in parameter λ and the simulation length at each λ vary when the total length of nonequilibrium transitions changes (Table 2), with different numbers of energy derivatives stored. For a 250 ps nonequilibrium transition (see Table 2), for example, we can use different combinations of λ interval (0.02, 0.01, 0.004) and simulation length (5, 2.5, and 1 ps) at each λ value, with 51, 101, and 251 energy derivatives stored, respectively. The pmx script analyze_dhdl.py^28^ with BAR estimator was used to obtain the free energy difference estimations.

**Table 2:** Statistical Metrics of the Predicted Binding Free Energies (kcal/mol) When the Length of the Nonequilibrium Transition Is Varied^a^

simulation length	MUE	MSE	RMSE	*r*	length per λ (ps)	failure (%)^b^
2 ns (Δλ = 0.002)	0.87(0.09)	0.08(0.09)	1.12(0.11)	0.51(0.09)	4	0
1 ns (Δλ = 0.002)	0.84(0.09)	0.07(0.09)	1.09(0.11)	0.51(0.09)	2	0.1
500 ps (Δλ = 0.004)	0.85(0.09)	0.11(0.09)	1.11(0.10)	0.51(0.09)	2	0.1
250 ps (Δλ = 0.02)	0.94(0.11)	0.06(0.11)	1.24(0.14)	0.48(0.09)	5	0
250 ps (Δλ = 0.01)	0.85(0.09)	0.07(0.09)	1.08(0.11)	0.51(0.09)	2.5	0
100 ps (Δλ = 0.02)	0.86(0.09)	0.11(0.09)	1.12(0.12)	0.52(0.08)	2	0.1
50 ps (Δλ = 0.04)	0.83(0.09)	0.13(0.09)	1.09(0.11)	0.55(0.08)	2	0.5
500 ps (Δλ = 0.002)	0.95(0.11)	0.07(0.11)	1.24(0.12)	0.48(0.08)	1	8.3
250 ps (Δλ = 0.004)	1.30(0.16)	0.03(0.16)	1.78(0.23)	0.36(0.11)	1	20.9

a The 58 ligand pairs for BACE are used.

b The simulation ended prematurely without reaching the final (λ = 0 or λ = 1) state.

## Results

To assess the accuracy and precision of the nonequilibrium method, we evaluated the binding affinities of the 503 ligand pairs to their target proteins and compared the nonequilibrium results with those from the equilibrium simulations.^8^ The comparison was made for the full set of ligand pairs, while a much more extended study was performed for the 14 proteins with one randomly selected ligand pair each (Table S2).

There are many simulation parameters in nonequilibrium free energy calculations, of which some are general for any simulation, such as those within the chosen force field, the choice of protein/ligand/water models, and the settings for the simulations, while some others are specific to the nonequilibrium free energy method. Here we focus our investigation on the latter: they are key for the sufficiency of conformational sampling, including the number of replicas per ensemble (increased from 5 to 20), the length of each equilibrium simulation at the end points (1–10 ns), the length of each nonequilibrium transition (varying between 50 ps and 2 ns), and the number of snapshots used in each nonequilibrium calculation (increased from 100 to up to 2500). The total simulation time for a single fully extended nonequilibrium free energy calculation then reaches up to 40.4 μs, resulting from ensembles of 20 equilibrium replicas at each end point of 10 ns duration and 10,000 nonequilibrium transitions of 2 ns duration in each direction, there being two end points and two directions for the nonequilibrium transitions.

Based on our findings from these studies, we critically evaluate the relative performance of equilibrium and nonequilibrium RBFE with respect to the accuracy of the predictions and the computational cost. We formulate a set of recommendations for the optimal execution of nonequilibrium RBFE calculations. Finally, we investigate the nature of the distributions of the predicted binding free energies, which are generated from ensembles composed of a sufficiently large number of members.

### Comparison of Equilibrium and Nonequilibrium Methods

**Figure 2:**
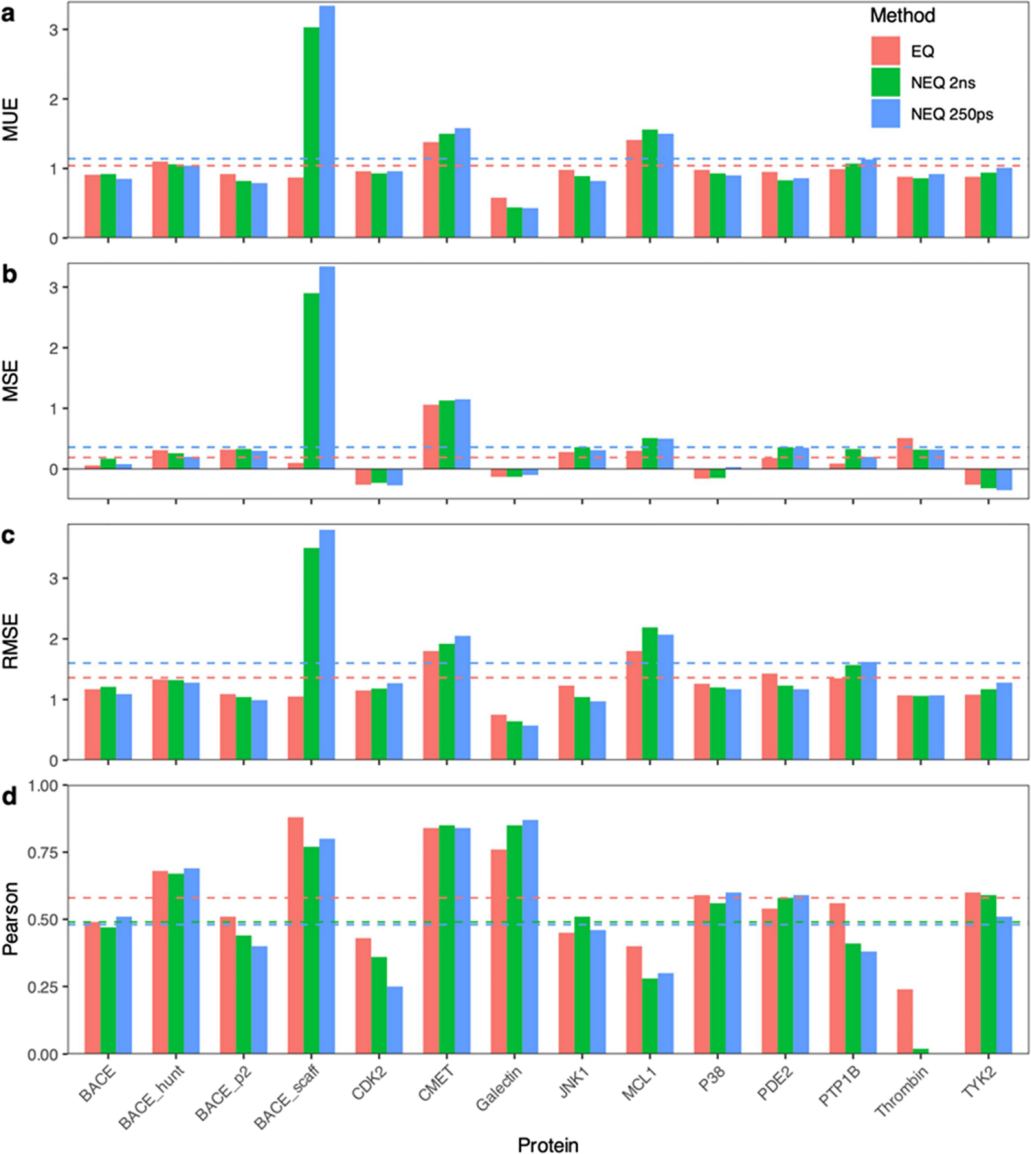
Performance of equilibrium (EQ) and nonequilibrium (NEQ) methods in free energy predictions. The NEQ results are generated from alchemical transitions with durations of 2 ns (NEQ 2 ns) and 250 ps (NEQ 250 ps). Four different metrics are used to assess the performance for each molecular system, including (a) mean unsigned error (MUE), (b) mean signed error (MSE), (c) root-mean-square error (RMSE), and d) Pearson correlation coefficient (*R*) between predicted binding affinities and experimental results. The metrics are also shown for all pairs of perturbations (dashed lines). The green and blue dashed lines from NEQ methods overlap with each other for MUE, MSE, and RMSE (Table S1). It is clear that the equilibrium method is more accurate and precise than the nonequilibrium one. All energies are in kcal/mol.

**Figure 3:**
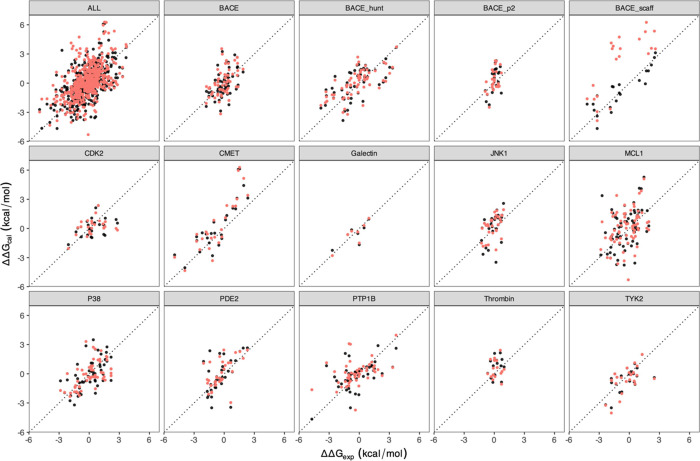
Comparison of predicted binding free energies with experimental measurements using equilibrium (black) and nonequilibrium (red) simulations. The comparison for the entire data set is presented in the upper left panel, while individual protein systems are depicted in separate panels. The dotted lines represent the line of perfect agreement (*y* = *x*).

The predicted RBFEs, from equilibrium simulations in our previous study^8^ and from nonequilibrium simulations in the current study, are compared with one another and against available experimental data. The nonequilibrium results are generated with durations of 2 ns, 250, 100, and 50 ps (Tables S1 and S2) for the nonequilibrium transitions to study the effect of transition time on the accuracy and precision of the predictions. In general, the difference in the length of nonequilibrium transitions does not produce statistically significant differences in the statistical metrics (Tables S1 and S2). However, certain cases, such as BACE_scaff in Table S1 and BACE_scaff and MCL1 in Table S2, do exhibit significant differences between nonequilibrium runs with durations of 250 and 100 ps. Not surprisingly, longer nonequilibrium transitions yield results that more closely align with those from equilibrium simulations (Table S2). As we recommend a duration of 250 ps for the nonequilibrium transition (see details in the Results section), we focus here on the comparison of nonequilibrium approach with this setting and the equilibrium method (Figure 2 and Table S1) for each protein target, and for all ligand pairs. For the entire data set, the nonequilibrium method generates larger mean unsigned errors (MUEs), larger mean signed errors (MSEs), larger root-mean-square errors (RMSEs), and a lower Pearson correlation coefficient than the equilibrium method (Figure 2 and Table S1). For individual protein targets, however, the differences between the two methods are difficult to discern visually (Figures 2 and 3) and are not statistically significant (Table S1) for all systems but BACE_scaff. BACE_scaff performs significantly worse using the nonequilibrium method in terms of the metrics MUE, MSE, and RMSE (discussed below). Even excluding this molecular system, the comparison between the two methods still reveals slightly lower overall MUE, MSE, and RMSE values, as well as a slightly higher Pearson correlation coefficient for the equilibrium method, although the differences are no longer statistically significant.

The nonequilibrium results for BACE_scaff are significantly worse than those from the equilibrium simulations, with MUE, MSE, and RMSE of 3.34, 3.34, and 3.80 kcal/mol from the nonequilibrium method (using 250 ps nonequilibrium transitions) and 0.87, 0.10, and 1.05 kcal/mol from the equilibrium method, respectively (Figure 2 and Table S1). The results from the nonequilibrium method do not improve much on increasing the duration of transitions to 2 ns, with the statistical metrics only slightly improving to 3.03, 2.90, and 3.50 kcal/mol (Table S1). The predictions from the complex leg (Figure 1a) are primarily responsible for this degradation (Figures S1 and S2). The BACE_scaff system was designed to investigate free energy predictions for more challenging scaffold modifications^43^ where the compound pairs have heterocycles of varying sizes. Compared to R-group modifications, scaffold hopping is more challenging in alchemical free energy calculations, especially for single-topology alchemical implementation where a soft bond stretch potential is needed.^44^

The TIES protocol^45^ uses a modified dual topology approach within which hybrid compounds are much more straightforward to construct. The dual topology method does not introduce geometrical strain from redundant degrees of freedom of dummy atoms, while the single topology method does and hence may bias the free energy results.^46^ In the dual topology, the rings of varying sizes in the compound pair can be easily represented as the appearing and disappearing groups in the alchemical region, along with any adjacent R-groups, of which the conformation may differ in the two compounds. The strategy generates more atoms in the alchemical region than when a single topology is used. Because of the modifications at the scaffold, the BACE_scaff systems have significantly more atoms in the alchemical region, with an average of 42 atoms, while other systems have only about half the number of atoms on average (Figure S3).

The number of atoms in the alchemical region, referred to as the size of the alchemical region, plays an important role in the convergence of the free energy predictions. Our previous studies have shown that the precision of TIES predictions is inversely proportional to the size of the alchemical region.^8,45^ The interaction of the atoms in the alchemical region with the environment is scaled down in the intermediate λ states, making them more flexible and thus prone to high fluctuations in their energy derivatives, leading in turn to lower precision. The large size of the alchemical region for BACE_scaff requires a longer simulation time for the mixture to converge. In addition, the charged or polar groups in the BACE_scaff compounds also present a challenge for the convergence of conformational sampling.^8^

To examine the convergence of nonequilibrium transitions for this molecular system, we further increased their duration from 2 to 4 ns for each individual run. Significant improvements can be observed from the extended transitions, with MUE, MSE, and RMSE reducing from 3.03, 2.90, and 3.50 kcal/mol at 2 ns to 2.01, 1.96, and 2.24 kcal/mol when the simulation length is doubled (Figure S4). Even with the extension, the statistical metrics remain much worse than those from the equilibrium simulations (Table S1 and Figure S4), albeit the total computational cost for each nonequilibrium calculation is significantly higher than that for each equilibrium calculation. A single nonequilibrium calculation using a transition length of 2 and 4 ns amounts to 500 and 900 ns of total production runs, respectively, compared with 260 ns required for a single equilibrium calculation. Long durations of nonequilibrium transition were also found to be required for some cases in a study of binding affinity changes of protein–protein complex due to mutations,^30^ being up to 8 ns, while 1–2 ns was sufficient for most mutations. The improvement in accuracy can be attributed to the increased closeness of the forward and reverse work distributions in the case of longer nonequilibrium transitions (Figure S5), resulting from smaller work dissipation along the alchemical path as compared to shorter transitions. The overlap between the two distributions, however, does not manifest a significant increase. The lack of convergence of the predictions for this molecular system highlights a problem with the commonly used “fast transitions” approach in nonequilibrium simulations, which must be approached with considerable caution when the alchemical region is large.

### Effect of Overlap between Work Distributions on Accuracy and Precision

We have used different durations for the nonequilibrium transitions in the complex leg (Figure 1a) for the entire data set. The simulations that conform to our recommendation (see below) – an ensemble of 5 equilibrium runs at the end points with 10 ns each, and 100 nonequilibrium transitions for 250 ps – generate binding free energy differences which are comparable to, or slightly better than, those from 2 ns long nonequilibrium transitions, although the differences are not statistically significant (Figure 2 and Table S1). This is counterintuitive, and certainly fortuitous, as shorter simulations usually lead to degraded accuracy. To locate the reasons for this, we investigate the overlaps of the forward and reverse work distributions from 250 ps and 2 ns nonequilibrium transitions, and their effects on the accuracy and uncertainty of the predicted binding free energies.

Although it has been widely acknowledged that the calculated free energies from nonequilibrium methods are affected by the lack of overlap between the forward and reverse work distributions,^28^ no comprehensive analysis has been conducted to establish a quantitative or even a qualitative correlation between the accuracy and precision of predictions and the degree of overlap. To quantify the degree of overlap, we define an overlap coefficient as the area of intersection of the two probability density functions; it offers a simple way to evaluate the similarity or difference among samples collected from the forward and reverse transitions. In our current data set, we observe significant variations in the ease of achieving substantial overlap among different molecular targets (Figure S7) or ligand pairs. Specifically, the BACE_scaff systems within the data set exhibit no overlap in most cases using 250 ps or 2 ns transition lengths in a protein environment, while TYK2 complexes generally display substantial overlap in the same setting. Moreover, the overlap coefficient varies significantly across different ligand pairs binding to the same protein. For instance, in the case of JNK1, the overlap coefficient demonstrates the largest range, varying from 0.03 to 0.94 across 31 ligand pairs for 250 ps transitions and from 0.01 to 0.96 for 2 ns ones.

**Figure 4:**
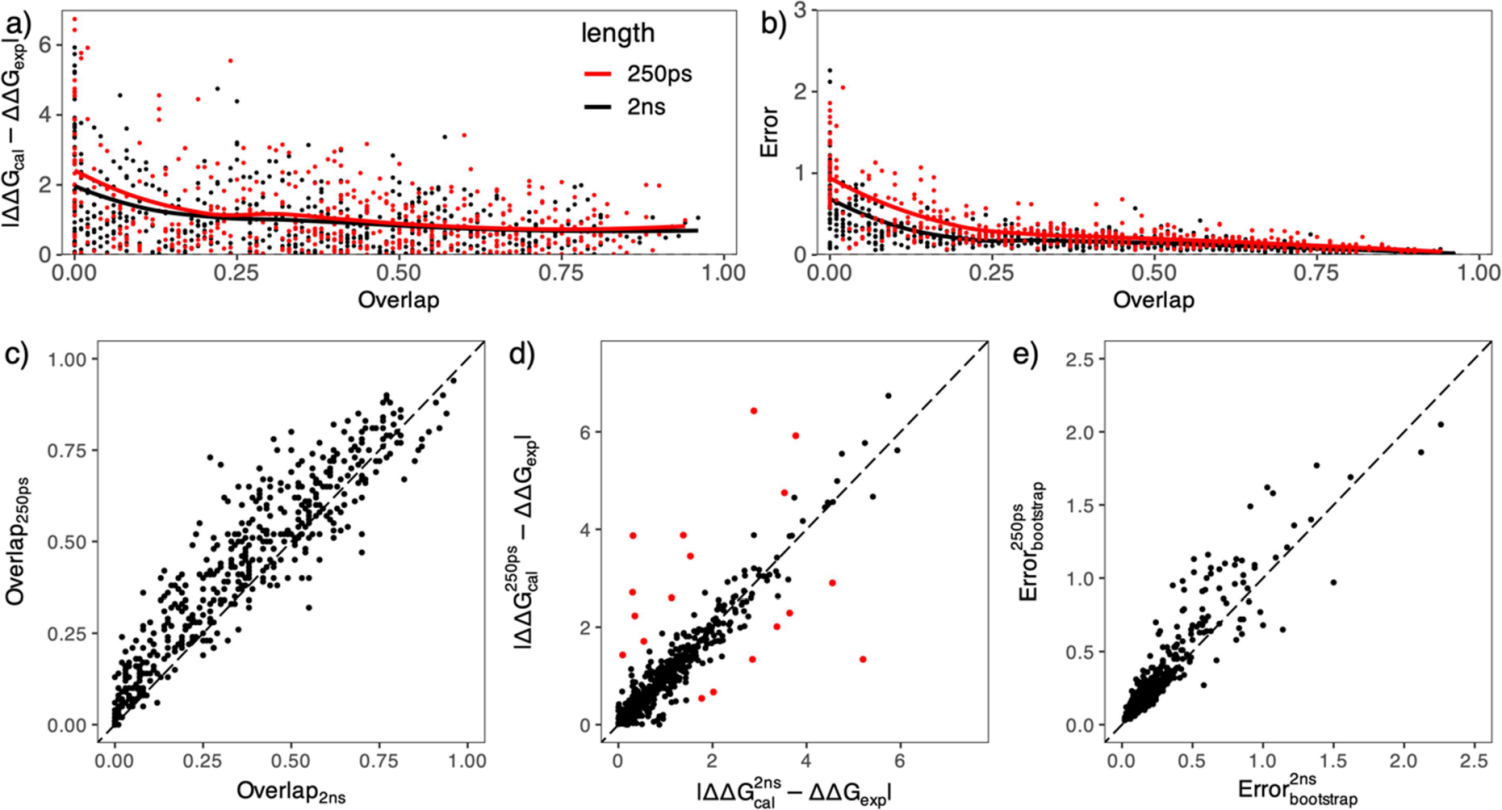
Impact of work distribution overlap on the accuracy and precision of the predicted free energy changes. The overlap is calculated as the intersection of the areas under the probability distribution functions of the forward and reverse work values from the complex simulations. The accuracy (a), measured by unsigned error |ΔΔ*G*_cal_ – ΔΔ*G*_exp_|, and the precision (b), quantified by the errors from the bootstrap approach, are both inversely related to the overlap for predictions from 2 ns (black dots) and 250 ps nonequilibrium transitions. LOESS (locally estimated scatterplot smoothing) regression (black and red lines) is used to display the trends clearly. While 2 ns transitions generally reduce the overlap (c), they do not evidently affect the accuracy (d); however, they typically lead to a reduction in bootstrap errors (e). The red dots in (d) indicate the predictions in which the differences between the 2 ns and 250 ps durations are larger than 1 kcal/mol. All energies are in kcal/mol.

Our findings demonstrate that both the accuracy and precision of the calculations exhibit an inverse relationship with the overlap coefficients (as depicted in Figure 4a,b). When little to no overlap is present, the predictions become noticeably less accurate and are accompanied by significantly larger uncertainties. When using 250 ps transitions, there are 30 ligand pairs with zero overlap, 19 of them (63.3%) having an accuracy (|ΔΔ*G*_cal_ – ΔΔ*G*_exp_|) worse than 2 kcal/mol; those with overlap between 0 and 0.5, 53 out of 279 pairs (19.0%) have an accuracy worse than 2 kcal/mol; when the overlap >0.5, only 5 out of 194 pairs (2.6%) have an accuracy worse than 2 kcal/mol. Some apparently “accurate” predictions with zero overlap are doubtless purely a matter of chance. The BAR implementation of CHARMM employs a ≥1% overlap as a guideline for generating free energy predictions.^47^ However, our analysis in Figure 4a indicates that the absence of overlap does not lead to an abrupt increase in the inaccuracy of predicted binding free energy differences. Therefore, we would recommend that a warning message be issued instead of rejecting the free energy estimate^47^ when the overlap is low or absent.

The work distributions have a wider spread from short simulations, resulting in a larger range of work values (Figures S5 and S6) and consequently a larger uncertainty from bootstrap analysis (Figure 4e). In long nonequilibrium simulations, work dissipation is lowered, resulting in narrow work distributions from both the forward and backward transitions, usually with the peaks getting closer and the tails shrinking at both sides. When there is little or no overlap in the 250 ps simulations, the shrinking of the adjacent tails renders the overlap even less in the 2 ns simulations (Figure 4c and Figure S6). In situations where there is already sufficient overlap in the short simulations, the coalescence of the peaks that arise in longer simulations counters the effect from the shrinking of the adjacent tails (Figure S6), typically resulting in lower degrees of overlap (Figure 4c). The numbers of ligand pairs with zero overlap and overlap between 0 and 0.5 increase to 43 and 313, respectively, in the 2 ns transitions. However, the decrease in the overlap does not necessarily degrade the accuracy or precision of the predictions (Figure 4d,e), as the low overlap from the 2 ns simulations generally generates less inaccurate and less imprecise results than that from 250 ps simulations with the same level of overlap (Figure 4a,b). Based on the above analysis, we recommend against extending the duration of nonequilibrium transitions beyond 250 ps for cases with significant overlaps between forward and reverse work distributions. If sampling issues persist for such cases, one should consider adjusting other nonequilibrium parameters (see our recommendations below). Having said that, there are certainly cases (mostly with zero or negligible overlaps) where longer transitions may be beneficial, as we have shown above for the BACE_scaff system.

### Simulation Parameter Settings in the Nonequilibrium Method

There are a few simulation parameters that affect the accuracy and precision of the predictions and the computational cost of the nonequilibrium free energy calculations. These quantities are

(i) The number of replicas of the equilibrium simulations at the two end points.
(ii) The length of each equilibrium simulation at the end points.
(iii) The number of nonequilibrium transitions.
(iv) The length of each nonequilibrium transition.

The first two parameters determine the quality of conformational sampling at the end points, which is critical for the accuracy of the predictions from the nonequilibrium method. The last two parameters concern the nonequilibrium transitions, which are important for the precision as well as the accuracy of free energy predictions. To investigate the settings for these parameters, we extended the simulations substantially for a subset of the molecular systems (Table S2) and performed a thorough analysis of their impact on the predictions. In the two legs of the alchemical process in the thermodynamic cycle (Figure 1a), the simulations of the compound pairs in the protein environment (complex leg) are extremely demanding in computational terms. We therefore focus only on this step for the extended studies. Aldeghi et al. have also evaluated different nonequilibrium protocols for the prediction of binding affinity changes upon protein mutations using GROMACS.^29^ We will compare our evaluations to theirs whenever possible.

#### Number of Replicas at the End Points

**Figure 5:**
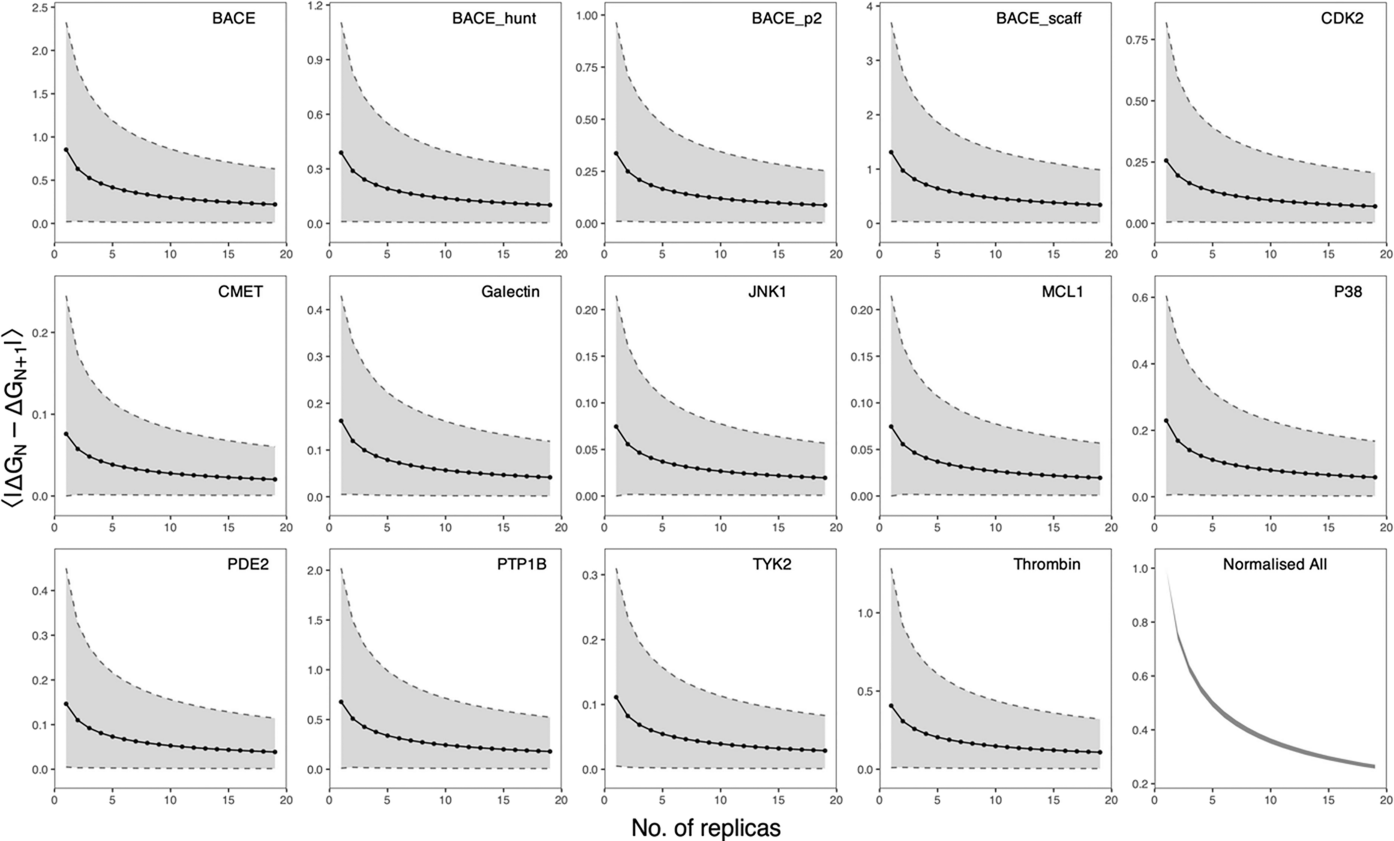
Average impact of an extra replica on the computed Δ*G*. For each ligand-protein system, 20 free energies are calculated with the nonequilibrium approach using only a single equilibrium replica at each end point. Δ*G*_*N*_ and Δ*G*_*N*+1_ are averages of *N* and *N* + 1 free energies randomly selected from the data set. The bootstrapped average absolute differences of Δ*G*_*N*_ and Δ*G*_*N*+1_ are shown as black dots and lines with the 95% confidence intervals shown as shaded regions. The bottom-right panel shows the relative changes of ⟨|Δ*G*_*N*_ – Δ*G*_*N*+1_|⟩ for all complexes, normalized by ⟨|Δ*G*_1_ – Δ*G*_2_|⟩; the apparent single line is the result of highly overlapped lines from all systems. All *y*-axes are energies in kcal/mol, except the one in the bottom-right panel, which is unitless.

We investigate the impact of using different numbers of replicas at the end points on the computed Δ*G* using the nonequilibrium approach. For this, a data set with 20 predicted free energies is used, each computed using a single equilibrium replica at both end points and one hundred individual nonequilibrium transitions in each direction starting from frames extracted from the equilibrium replica at the corresponding end points. We measure the magnitude of change in Δ*G* values with each added replica going from ensemble size 1 to 20. The results are displayed in Figure 5. The absolute change in Δ*G* is calculated by using bootstrapping. This method involves resampling with replacement *N* (1 ≤ *N* ≤ 19) and *N* + 1 input data points to calculate one value of |Δ*G*_*N*_ – Δ*G*_*N*+1_|. This process is repeated many times (in our case, 100,000 times) and the statistics of interest for each bootstrap population is calculated. The 95% confidence intervals, or 2.5 and 97.5% percentiles, are used to provide an estimate of the uncertainty associated with the bootstrapped averages (Figure 5). We select this metric because the bootstrap sample distributions are not normal, as indicated in Figure 5 by the asymmetrical positioning of the upper and lower limits relative to the mean.

A single replica is shown to be unreliable. Introducing a second replica results in an average Δ*G* change of approximately 0.38 kcal/mol, with variations ranging from 0.07 kcal/mol for JNK1 and 1.31 kcal/mol for BACE_scaff (Figure 5). The impact is system-dependent: when proteins are structurally stable and the size of the alchemical regions is minimal, adding more replicas has a negligible impact on the final value, while in other cases, adding the second replica can have a significant impact on the computed values, by a change up to 1.31 kcal/mol for the BACE_scaff case. When using five replicas, adding a sixth achieves only limited gains, with an average change of 0.19 kcal/mol; 10 out of 14 molecular systems investigated have changes less than 0.19 kcal/mol, while all but one have changes less than 0.42 kcal/mol. Importantly, all molecular systems demonstrate consistent relative changes when an additional replica is added, although the absolute changes may vary significantly (Figure 5). This characteristic allows for the estimation of the relative impact that an extra replica can have before conducting additional simulations. One can then set a threshold for the error bars and estimate how many replicas will be required for the threshold to be achieved. The BACE_scaff system, for example, requires an ensemble of 12 replicas to reach a change of less than 0.42 kcal/mol when an extra replica is added. For general practice, we recommend a choice of five replicas for the equilibrium simulations at the end points, which is a good trade-off between the computational cost and the uncertainties in the predictions. This is consistent with the equilibrium approach, where an ensemble of five replicas is found to be an optimal choice at all λ values.^7^ Aldeghi et al.^29^ did not evaluate the ensemble size in their study, but coincidentally used 5 “repeated” equilibrium simulations as we suggested here.

#### Duration of Equilibrium Runs at the End Points

**Figure 6:**
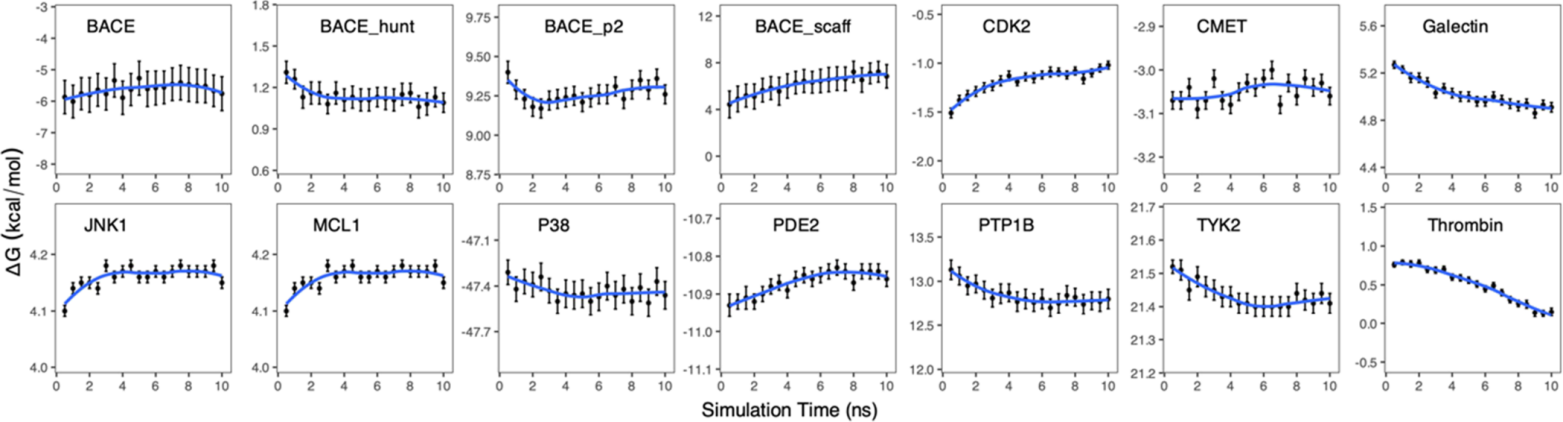
Predicted free energy changes from nonequilibrium simulation for compound pairs in the ligand-protein complex. Each data point at simulation time *t* is calculated using 100 individual nonequilibrium runs for each direction, with snapshots extracted evenly from the equilibrium simulations from 0 to *t*. LOESS (locally estimated scatterplot smoothing) regression (blue lines) is used to display the trends clearly.

While the ensemble approach is the only reliable way to sample conformations, a sufficient duration is evidently also required for the equilibrium runs at the end points to ensure that the end point states of the molecular systems have been sufficiently sampled. To examine the dependence of the predictions on the length of equilibrium simulations, we calculated the time-dependent ensemble average of the predicted free energy changes ⟨Δ*G*(*t*)⟩ for compound pairs in the protein environment (Figure 6). Each ⟨Δ*G*(*t*)⟩ is calculated from the nonequilibrium runs with 100 snapshots evenly extracted from equilibrium simulations from 0 to *t*. Almost all of the molecular systems exhibit relatively large changes when using trajectories from the first 5 ns, which either plateau or diminish when the trajectories extend beyond 5 ns. There are, however, cases indicating that longer equilibrium simulations are required. The BACE_scaff system, which we have discussed above, needs longer equilibrium runs at the end points. Thrombin is another protein that also needs longer equilibrium simulations. Slow convergence has been reported previously for this system,^48^ since ligands may occupy multiple conformations due to a ring flip in the S1 pocket of the protein, and owing to the populations of two conformations of another ring which moves in and out of the S3 pocket. There may be large energy barriers separating the different conformations, requiring long equilibrium simulations at the end points for the ligands to sample other conformations than the starting one. The nonequilibrium transitions, however, converge relatively fast due to the small and rigid alchemical region (a fluorine atom being replaced by an ethyl group); the small error bars in the predicted free energy changes (Figure 6) indicate that the nonequilibrium runs are well behaved. Because of the crucial importance of the equilibrium sampling on the accuracy of the free energy prediction, we recommend the use of a 10 ns equilibrium run as a practical rule of thumb to start, followed by a stepwise increase in the simulation length only for those molecular systems for which convergence is not achieved. Longer simulation times should only be required for systems where slow convergence is encountered due to sluggish conformational interconversions. Such a progressive increase in simulation length would ensure the optimal use of computational resources while achieving the desired level of accuracy. This is quite different from what has been indicated by Aldeghi et al.^29^ who did not find strong associations between the length of the equilibrium simulations and the accuracy or precision of the free energy calculations (probably because they were only studying protein mutations which are relatively less complex than ligand transformations), and sequentially used 3 ns each for the five equilibrium simulations they performed. A study on protein–protein binding free energy calculations, however, has used 40 ns for the equilibrium simulations at the end points, and concluded that the accuracy of the nonequilibrium predictions could be improved via longer equilibrium simulations.^30^ It should be noted that, while enhanced sampling techniques such as replica exchange with solute tempering (REST2)^49^ have been used to accelerate conformational sampling, these techniques offer no guarantee of improving the accuracy of binding free energy predictions.^8^ We therefore do not recommend the use of such an enhanced sampling technique for the equilibrium simulations at the end points.

#### Number of Simulations for Each Nonequilibrium Transition

**Figure 7:**
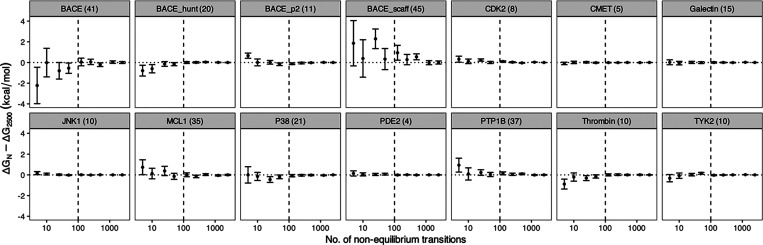
Convergence of the predicted binding free energy change Δ*G*_*N*_ from nonequilibrium runs consisting of *N* individual simulations. In line with our recommendation for the nonequilibrium method, an ensemble of 5 replicas is used at each end point, with 10 ns equilibrium simulation each; the duration of the nonequilibrium transition is 250 ps. The *N* simulations are uniformly selected from the total 2500 nonequilibrium transition runs. The numbers in parentheses after the protein names are the numbers of atoms in the alchemical region. The vertical dashed line is *N* = 100.

To investigate the convergence of free energy predictions on the number of nonequilibrium transitions in each nonequilibrium calculation, we selected different numbers of nonequilibrium transitions going up to 2500 from an ensemble of 5 end point replicas. The choice of 5 instead of all 20 replicas is based on our recommendation above on the number of replicas at the end points. As recommended above, a duration of 10 ns is used for each equilibrium run. A length of 250 ps, as recommended below, is used for each nonequilibrium transition. Free energy changes Δ*G*_*N*_ are calculated using *N* transitions uniformly selected from 2500, and compared with that using all 2500 transitions, Δ*G*_2500_ (Figure 7). The predicted free energy changes and their uncertainties converge after using 100 transitions in a single nonequilibrium calculation for all but one molecular system investigated (Figure 7). The exception is BACE_scaff, which, as we have discussed above, has the largest number of atoms growing in or growing out during the alchemical transformation (Table S2). We discuss this exception in more detail below, but in general, we recommend using at least 100 nonequilibrium transitions for each calculation. This is consistent with what Aldeghi et al.^29^ have recommended, who found that the precision and accuracy of the predictions improved quickly from 10 to 100 nonequilibrium transitions, after which further improvements came at a higher cost.

#### Simulation Duration of Nonequilibrium Transitions

We have performed nonequilibrium transitions with different simulation lengths, from 50 ps to 2 ns, for the 58 ligand pairs of the BACE system. During alchemical transitions, particularly those involving a large number of atoms growing in the sample, simulations may necessitate a considerable amount of time to reach equilibrium. Consequently, when the nonequilibrium transition is very brief, it is not unexpected that some simulations become unstable and terminate prematurely. The choice of soft-core potentials^9^ also affects the stability of the integrators used and is likely to contribute to the failure rate of the simulations when the duration of nonequilibrium transition is very short. Our simulations with different lengths show that the nonequilibrium transition with NAMD requires no less than 2 ps at each λ value to render the molecular system stable; otherwise, a significant number of simulations typically fail (last two rows in Table 2), resulting in less than 100 successful nonequilibrium transitions and hence large RMSE and MAE values (Table 2 and Figure 7).

When the total length for the nonequilibrium transition simulation is fixed and the simulation length for each λ value is no less than 2 ps, the accuracy of the simulations is independent of the choice of the λ interval (Δλ), as evident from Table 2. There is no clear dependence of the accuracy on the nonequilibrium simulation length: as long as the simulations at each λ value are no shorter than 2 ps, the predicted binding free energy differences are comparable and the differences in the statistical metric are not statistically significant (Table 2). To ensure the conclusion is applicable to the entire data set, we have performed nonequilibrium transitions with different simulation lengths (50 ps, 100 ps, 250 ps, and 2 ns) for all ligand pairs in the data set and calculated the statistical metrics for the predicted binding free energies (Table S1). Although the differences are again not statistically significant for all proteins but BACE_scaff, the averages of these statistical metrics from 250 ps are consistently better than those from 100 and 50 ps for the entire data set. The extension of simulations from 250 ps to 2 ns does not yield substantial improvements based on the comparison (Figure 2 and Table S1). In addition, more nonequilibrium transitions fail when the duration of these simulations is less than 250 ps, with failure rates of 0.8, 1.8, and 5.5% for 250, 100, and 50 ps runs, respectively. The failure rate is also system dependent: for the 50 ps runs, for example, some protein systems have failure rates up to 20.5%, while others have no failures at all. Taking into account all of these observations, we recommend 250 ps for the nonequilibrium transitions. This is much longer than the 80 ps length Aldeghi et al.^29^ have recommended, who evaluated the lengths of the nonequilibrium transitions only up to 100 ps, and concluded that there are no more benefits in terms of accuracy when increasing the lengths beyond 80 ps.

The guidelines we have outlined above should facilitate large-scale applications of the nonequilibrium method for relative binding free energy calculations. However, there are always cases in which care has to be taken. The large size of the alchemical region, for example, requires a longer nonequilibrium transition time to obtain converged results. A poor overlap between the work distributions from the two directions (Figures S5–7) can be due to the short transition time and/or the large differences in the appearing and disappearing regions of the ligand (Figure 7 and Table S2). Such differences come not only from the number of atoms but also from the lack of mapping between atoms in the appearing and disappearing groups where the scaffold is modified. When the changing atoms in the two end points have a good one-to-one mapping, the conformational space will overlap substantially. Even when the number of atoms is not small, this may not lead to large uncertainties. This is the case for the compound pair of MCL1. Although there are 17 and 18 atoms disappearing and appearing (Table S2), respectively, they have a good one-to-one mapping between the two groups of atoms. The calculated free energy changes therefore converge quickly without a large number of nonequilibrium transitions (Figure 7).

### Distribution of the Free Energy Changes from Nonequilibrium Method

Our previous studies have shown that non-Gaussian distributions are present in binding free energies for a considerable percentage of ligand-protein systems, from both MD simulations and experimental measurements.^39^ Significantly more “outliers” can be produced from these non-Gaussian distributions than one would anticipate were the statistics to conform to a normal distribution. Ensemble simulations with a sufficiently large number of replicas are required to extract reliable statistics in such cases. The extension of the number of replicas to 20 enables us to investigate the distributions of the RBFE free energy predictions in the case of the nonequilibrium method. The extension has been done for the step where the ligand interactions are turned on or off in the protein environment (Figure 1), as the main contribution to the uncertainty comes from this step.

**Figure 8:**
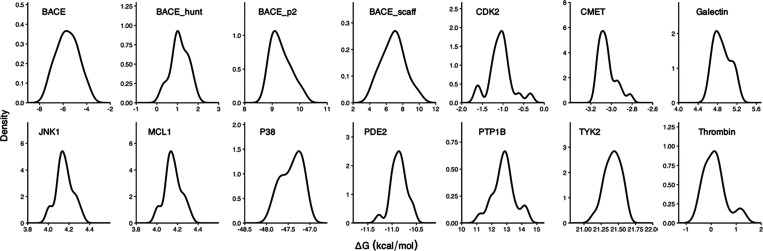
Distributions of free energy changes in the complex leg of the thermodynamic cycle. Twenty sets of nonequilibrium simulations are performed, with snapshots extracted from individual equilibrium runs at the end points.

The probability plots (Figure 8) show clearly that some distributions are skewed and asymmetric, with fat tails or small peaks on one or both side(s). The existence of multiple modes in the calculated free energy changes indicates the presence of multiple conformations for one or both ligand(s). The nonequilibrium work values are also skewed and asymmetric (Figures S5 and S6). While the BAR estimation routine itself makes no assumptions about the shape of the work distributions,^28^ the Crooks’ fluctuation theorem and Jarzynski’s equality make use of an exponential average, which is highly sensitive to the tails of the work distribution. Sufficient sampling is needed to ensure robust statistics for work distributions.

The distributions of free energy changes show that, for the same molecular system, the predictions obtained from two independent nonequilibrium simulations (independent equilibrium simulations at the end points and nonequilibrium transitions from each equilibrium run) can vary by up to 10 kcal/mol, a range similar to that reported from equilibrium methods^6^ for binding of small molecules to proteins. Such variations are much larger than the experimentally observed maximum binding free energy difference of the inhibitors under investigation. Only ensemble-based studies can effectively explore the relevant parts of phase space, namely, the conformations at the two end points in the case of nonequilibrium simulations. The magnitude of the estimated error decreases by increasing the number of replicas in an ensemble simulation, directly providing information on the convergence of the results (Figure 5).

## Conclusions

We have presented a systematic investigation of the nonequilibrium method for relative binding free energy predictions. We have used ensemble simulations as they are essential owing to the chaotic mixing properties of MD systems.^6^ With a large data set of over 500 compound pairs binding to a diverse set of 14 different protein targets, we have demonstrated that the nonequilibrium method generates results comparable to the equilibrium method. The computational cost for the former can be less if the protocol is carefully designed, but the wall clock time is usually longer than the latter. For the equilibrium approach with TIES, employing an ensemble size of 5, a production run of 4 ns and 13 λ windows, one requires a total of 260 ns production simulations, while the nonequilibrium approach, with 5 replicas at the end points for 10 ns runs and 100 nonequilibrium runs for 250 ps in each direction, needs 150 ns in total (Table 1). There are, however, cases that require a much longer duration for the nonequilibrium transition, such as the BACE_scaff system studied here and certain mutations reported in the literature.^30^ All simulations in the equilibrium approach can be carried out concurrently while, in the nonequilibrium approach, the nonequilibrium runs for transferring one state to another depending on a pre-existing equilibrium state having been established at the end points. The two sets of runs were performed sequentially. The equilibrium approach therefore produces results within a shorter wall clock time on modern supercomputers, an important consideration if we wish to produce actionable results from such simulations.

Compared with the equilibrium method, the nonequilibrium approach may require less computational cost, but it certainly entails greater computational complexity. There are more “moving parts”—including additional simulation parameters to be selected—in the method, which affect both the accuracy of its predictions and the computational cost, as enumerated in the following. (1) The conformations at the end points need to be sufficiently sampled. (2) The work values from nonequilibrium runs are non-normally distributed, affecting the overlap of work distributions from the nonequilibrium runs in the forward and reverse directions. (3) Insufficient overlap between the work distributions from the two directions makes the calculations less accurate. (4) The overlap criteria, which indicate whether the nonequilibrium method is reliable, themselves demand the use of ensembles. (5) When the difference between the two physical end points is large, the conformations at the end points need to be sufficiently well sampled, and the nonequilibrium transitions need to be of sufficient duration to ensure that the predicted free energy changes are converged. (6) Just as for the equilibrium approach, we recommend using a flexible protocol with the nonequilibrium approach too by adapting all or some of these parameters depending on the system using our guidelines. (7) One significant practical limitation specific to the nonequilibrium approach is that the length of the nonequilibrium transitions invoked cannot be simply extended; if one wishes to increase it, *the entire set of transitions needs to be rerun*, which hampers its flexibility and is highly inconvenient during large-scale applications.

We have confirmed that the distribution of free energies obtained from independent replica simulations exhibits deviations from the Gaussian behavior usually assumed. Just as we have demonstrated numerous times with the equilibrium method, performing ensemble simulations is also crucial for the nonequilibrium method. One implication of the non-normal statistics observed here is that the mean of the quantities of interest does not coincide with the peak in the distribution of that quantity. An important consequence of such non-normality is that the ensemble size cannot be arbitrarily small. We investigated these distributions for the calculated free energy changes by extending the number of replicas at the end points to 20. In practice, however, we find that 5 membered ensembles are usually sufficient.

The detailed and systematic analysis of the settings of the simulation parameters enables us to make definitive recommendations for the implementation of the method to minimize the loss of accuracy and precision of the results obtained while keeping computational costs minimized. We recommend that ensembles be used with a minimum ensemble size of five for the equilibrium simulations at the end points for 10 ns. This is the same ensemble size that we have consistently and repeatedly recommended for the equilibrium method. For the step involving a nonequilibrium transition from one end point to the other, we recommend the use of 100 simulations with snapshots uniformly extracted from the five-replica trajectories at each end point for no less than 250 ps, which exceeds the typical temporal durations employed in many publications by two or three times. In cases where convergence is slow, such as for the BACE and BACE_scaff systems in the current study, prediction accuracy can be improved by increasing the ensemble size, extending the simulation time, and reducing the size of the alchemical region^8^ or a combination of these routes.

## Data Availability

The data underlying this study are available in the published article. All input structures and parameter files used in this study, along with experimental ΔΔ*G* values, are available at https://github.com/UCL-CCS/LargeScaleTIES.

## Supporting Information Available

Statistical metrics for the entire data set between the calculations and the experimental data and between the equilibrium and nonequilibrium methods, details of the subset of ligand-protein systems for extended simulations, comparisons of the free energy changes obtained from the equilibrium and nonequilibrium approaches in the complex leg and the solvent leg, reasons for the need of longer simulations for the BACE_scaff systems, overlap of work distributions from simulations with different durations, and work distributions from a very large number of nonequilibrium transitions (PDF)
