# Supplementary material for: Comparison of Equilibrium and Nonequilibrium Approaches for Relative Binding Free Energy Predictions
Source: J Chem Theory Comput. 2023 Oct 20;19(21):7846–60. doi: 10.1021/acs.jctc.3c00842 (PMC10653111; doi:10.1021/acs.jctc.3c00842)
Supplement: Supplementary file 1 — ct3c00842_si_001.pdf [file ct3c00842_si_001.pdf]

Supporting Information for

Comparison of Equilibrium and Non-Equilibrium Approaches for Relative Binding Free Energy Predictions

Shunzhou Wan[1], Agastya P. Bhati[1], Peter V. Coveney[1,2,3*]

[1]Centre for Computational Science, Department of Chemistry, University College London
[2]Advanced Research Computing Centre, University College London, London WC1H 0AJ, United Kingdom
[3]Computational Science Laboratory, Institute for Informatics, Faculty of Science, University of Amsterdam, Netherlands
*Author for correspondence, Email: p.v.coveney@ ucl.ac.uk

The main article describes a comparative study of equilibrium and non-equilibrium methods for computing relatively binding free energies. We have used a large dataset, comprising more than 500 ligand transformations spanning in excess of 300 ligands binding to a set of 14 diverse protein targets. The Supporting Information lists the statistical metrics MUE, MSE, RMSE and Pearson correlations for the entire dataset between the calculations and the experimental data (Table S1), and between the equilibrium and non-equilibrium methods (Table S2). We selected a subset of ligand-protein systems (Table S3) for extensive analysis, with the number of replicas for the equilibrium simulations extended to 20 and the number of non-equilibrium transitions to 10,000.

The comparison between the equilibrium and non-equilibrium approaches is made not only for the final $\Delta\Delta G$ (Figure 3 in the main text), but also for the free energy changes in the complex leg (Figure S1) and the solvent leg (Figure S2) in the thermodynamic cycle (Figure 1a). The size of the alchemical region is significantly larger for the BACE_scaff case than the other molecular systems (Figure S3), which requires longer duration non-equilibrium transitions to improve the accuracy of the predictions (Figure S4). One should note that longer simulations for the fast transition of one end point to the other typically do not increase the overlap of the work distributions from the forward and reverse transitions (Figures S5 and S6). Finally, to ensure the work distributions be considered representative, we present the distributions in Figure S7 with a large sample size, namely a total of 10,000 non-equilibrium transitions in each direction.

Table S1. The statistical properties for all the protein targets used in this study, which are calculated in comparison with experimental data. Mean unsigned error (MUE), mean signed error (MSE) and root-mean-squared error (RMSE) are in kcal/mol. Errors for MUE, MSE, RMSE, and Pearson correlation values from the bootstrapping approach are also reported. The non-equilibrium results are calculated with the same parameters of the simulation protocol except the lengths of the non-equilibrium transitions. The other parameters are: 5 replicas, each with a duration of 10 ns, for the equilibrium simulations at each of the endpoints, and 100 replicas for the non-equilibrium transitions between the two endpoints. The equilibrium method uses the standard TIES protocol in which 5 replicas are applied with 4 ns production runs at each of the 13 λ windows. The best statistical metrics (smallest absolute values for MUE, MSE and RMSE, and highest Pearson correlation) are shown in bold font.

	Equilibrium				Non-equilibrium (2 ns)				Non-equilibrium (250 ps)				Non-equilibrium (100 ps)				Non-equilibrium (50 ps)			
Protein	MUE	MSE	RMSE	Pearson	MUE	MSE	RMSE	Pearson	MUE	MSE	RMSE	Pearson	MUE	MSE	RMSE	Pearson	MUE	MSE	RMSE	Pearson
BACE	0.91±0.10	**0.06±0.15**	1.17±0.12	0.49±0.09	0.92±0.10	0.17±0.16	1.21±0.14	0.47±0.09	0.85±0.09	0.08±0.14	**1.09±0.10**	0.51±0.09	0.90±0.09	0.11±0.09	1.12±0.11	0.52±0.09	**0.83±0.09**	0.13±0.09	1.09±0.11	**0.55±0.08**
BACE_hunt	1.10±0.10	0.31±0.17	1.33±0.10	0.68±0.06	1.06±0.10	0.26±0.17	1.32±0.10	0.67±0.07	**1.04±0.10**	0.20±0.16	**1.28±0.10**	**0.69±0.06**	1.02±0.10	0.22±0.10	1.28±0.10	0.69±0.07	1.09±0.10	**0.20±0.10**	1.34±0.10	0.67±0.07
BACE_p2	0.92±0.11	0.32±0.20	1.09±0.11	**0.51±0.18**	0.82±0.13	0.33±0.19	1.04±0.14	0.44±0.14	**0.79±0.12**	0.30±0.19	**0.99±0.13**	0.40±0.15	0.81±0.12	0.26±0.12	1.01±0.13	0.43±0.16	0.85±0.12	**0.18±0.12**	1.04±0.14	0.43±0.16
BACE_scaff	**0.87±0.13**	**0.10±0.23**	**1.05±0.13**	**0.88±0.04**	3.03±0.38	2.90±0.43	3.50±0.35	0.77±0.07	3.34±0.40	3.34±0.40	3.80±0.38	0.80±0.07	4.47±0.41	4.37±0.41	4.85±0.40	0.80±0.07	4.52±0.44	4.52±0.44	4.95±0.45	0.75±0.08
CDK2	0.96±0.13	-0.26±0.22	**1.15±0.13**	**0.43±0.14**	**0.93±0.15**	**-0.23±0.23**	1.18±0.18	0.36±0.20	0.96±0.17	-0.27±0.25	1.27±0.20	0.25±0.23	0.97±0.16	-0.32±0.16	1.26±0.20	0.27±0.23	1.23±0.20	-0.36±0.20	1.59±0.24	0.01±0.25
CMET	**1.38±0.23**	**1.06±0.29**	**1.80±0.31**	0.84±0.04	1.50±0.24	1.13±0.31	1.92±0.30	**0.85±0.04**	1.58±0.26	1.15±0.34	2.05±0.34	0.84±0.04	1.48±0.24	1.09±0.24	1.91±0.32	**0.85±0.04**	1.72±0.26	1.23±0.26	2.16±0.28	0.78±0.06
Galectin	0.58±0.18	-0.13±0.28	0.75±0.24	0.76±0.31	0.44±0.17	-0.13±0.24	0.64±0.23	0.85±0.27	0.43±0.14	-0.10±0.21	0.57±0.16	0.87±0.28	**0.34±0.15**	-0.14±0.15	**0.53±0.21**	**0.90±0.23**	0.46±0.14	**0.03±0.15**	0.60±0.18	0.85±0.25
JNK1	0.98±0.13	**0.28±0.22**	1.23±0.17	0.45±0.17	0.89±0.10	0.36±0.18	1.04±0.10	0.51±0.15	0.82±0.09	0.31±0.16	0.97±0.09	0.46±0.15	0.76±0.09	0.32±0.09	0.91±0.08	**0.53±0.13**	**0.72±0.07**	0.39±0.07	**0.82±0.07**	0.46±0.13
MCL1	**1.41±0.13**	**0.30±0.21**	**1.80±0.18**	**0.40±0.12**	1.56±0.18	0.51±0.25	2.19±0.35	0.28±0.10	1.50±0.17	0.50±0.24	2.07±0.27	0.30±0.10	1.86±0.29	0.78±0.29	3.04±0.61	0.20±0.10	1.82±0.29	0.69±0.29	3.07±0.65	0.13±0.11
P38	0.98±0.11	-0.16±0.17	1.26±0.13	0.59±0.08	0.93±0.10	-0.15±0.16	1.20±0.13	0.56±0.09	**0.90±0.10**	**0.03±0.16**	**1.17±0.14**	**0.60±0.08**	1.02±0.11	0.12±0.11	1.31±0.13	0.52±0.09	1.36±0.16	0.67±0.16	1.82±0.20	0.45±0.09
PDE2	0.95±0.18	**0.18±0.24**	1.43±0.25	0.54±0.17	**0.83±0.15**	0.36±0.20	1.23±0.22	0.58±0.16	0.86±0.14	0.35±0.19	1.17±0.17	0.59±0.15	0.84±0.13	0.36±0.13	**1.15±0.16**	0.58±0.14	0.96±0.13	0.22±0.12	1.22±0.13	**0.69±0.11**
PTP1B	**0.99±0.13**	**0.09±0.19**	**1.35±0.17**	**0.56±0.14**	1.07±0.16	0.33±0.22	1.57±0.21	0.41±0.15	1.13±0.16	0.20±0.23	1.62±0.21	0.38±0.11	1.22±0.17	0.13±0.17	1.72±0.21	0.22±0.13	1.35±0.17	0.43±0.17	1.80±0.25	0.27±0.14
TYK2	**0.88±0.13**	-0.26±0.21	**1.08±0.16**	**0.60±0.13**	0.94±0.15	-0.32±0.23	1.17±0.17	0.59±0.13	1.01±0.16	-0.35±0.25	1.28±0.18	0.51±0.16	1.01±0.18	-0.38±0.18	1.34±0.24	0.46±0.18	1.00±0.17	**-0.25±0.17**	1.30±0.20	0.36±0.20
Thrombin	0.88±0.15	0.51±0.24	1.07±0.15	**0.24±0.23**	**0.86±0.16**	0.32±0.25	**1.06±0.15**	0.02±0.24	0.92±0.14	0.32±0.26	1.07±0.13	0.00±0.23	0.89±0.15	**0.31±0.15**	1.07±0.15	-0.00±0.24	0.95±0.17	0.36±0.17	1.16±0.16	0.02±0.24
All	**1.04±0.04**	**0.17±0.06**	**1.36±0.05**	**0.58±0.03**	1.14±0.05	0.36±0.07	1.60±0.06	0.49±0.04	1.14±0.05	0.36±0.07	1.60±0.08	0.48±0.04	1.26±0.07	0.44±0.07	1.92±0.15	0.40±0.05	1.34±0.07	0.53±0.07	2.02±0.16	0.37±0.05
All except BACE_scaff	**1.05±0.04**	**0.17±0.06**	**1.37±0.06**	**0.54±0.03**	1.06±0.04	0.25±0.07	1.46±0.09	0.49±0.04	**1.05±0.04**	0.23±0.06	1.43±0.07	0.50±0.04	1.12±0.06	0.27±0.06	1.68±0.17	0.42±0.05	1.20±0.06	0.36±0.06	1.78±0.17	0.40±0.06

Table S2. The statistical properties for all the protein targets used in this study, which are calculated by comparison with the equilibrium (EQ) and non-equilibrium (NEQ) methods. Mean unsigned error (MUE), mean signed error (MSE) and root-mean-squared error (RMSE) are in kcal/mol. Errors for MUE, MSE, RMSE, and Pearson correlation values from the bootstrapping approach are also reported. The simulation parameters are the same as these listed in the caption of Table S1.

	EQ vs NEQ (2ns)				EQ vs NEQ (250ps)				EQ vs NEQ (100ps)				EQ vs NEQ (50ps)			
Protein	MUE	MSE	RMSE	Pearson	MUE	MSE	RMSE	Pearson	MUE	MSE	RMSE	Pearson	MUE	MSE	RMSE	Pearson
BACE	0.42±0.09	-0.11±0.09	0.78±0.27	0.83±0.11	0.36±0.04	-0.03±0.04	0.45±0.04	0.94±0.02	0.42±0.05	-0.06±0.05	0.55±0.07	0.91±0.03	0.44±0.04	-0.07±0.04	0.55±0.05	0.91±0.02
BACE_hunt	0.32±0.05	0.05±0.05	0.50±0.09	0.94±0.02	0.29±0.04	0.11±0.04	0.43±0.07	0.96±0.02	0.31±0.04	0.09±0.04	0.42±0.04	0.96±0.01	0.31±0.03	0.11±0.03	0.40±0.04	0.97±0.01
BACE_p2	0.48±0.11	-0.01±0.11	0.75±0.15	0.79±0.10	0.49±0.12	0.03±0.12	0.77±0.15	0.76±0.10	0.56±0.12	0.07±0.13	0.84±0.16	0.73±0.13	0.62±0.11	0.14±0.11	0.84±0.14	0.74±0.11
BACE_scaff	3.01±0.42	-2.80±0.42	3.56±0.40	0.69±0.10	3.31±0.47	-3.24±0.46	3.93±0.40	0.68±0.09	4.33±0.50	-4.27±0.51	4.91±0.45	0.71±0.06	4.42±0.50	-4.42±0.50	4.97±0.47	0.66±0.10
CDK2	0.55±0.10	-0.03±0.10	0.73±0.11	0.65±0.20	0.56±0.09	0.01±0.09	0.72±0.11	0.66±0.20	0.59±0.09	0.07±0.09	0.73±0.10	0.65±0.20	0.72±0.12	0.10±0.12	0.93±0.12	0.56±0.23
CMET	0.37±0.04	-0.07±0.04	0.44±0.05	0.99±0.00	0.51±0.07	-0.10±0.07	0.62±0.08	0.98±0.01	0.42±0.05	-0.03±0.05	0.50±0.05	0.98±0.01	0.70±0.12	-0.18±0.13	0.93±0.18	0.94±0.03
Galectin	0.19±0.06	0.00±0.06	0.25±0.08	0.98±0.02	0.16±0.08	-0.02±0.08	0.26±0.09	0.97±0.02	0.28±0.08	0.01±0.08	0.35±0.06	0.95±0.08	0.20±0.06	-0.16±0.06	0.26±0.06	0.98±0.02
JNK1	0.25±0.05	-0.08±0.05	0.38±0.07	0.97±0.01	0.30±0.07	-0.03±0.06	0.47±0.11	0.96±0.01	0.33±0.08	-0.04±0.08	0.55±0.16	0.93±0.03	0.60±0.12	-0.11±0.12	0.88±0.19	0.79±0.07
MCL1	0.79±0.18	-0.20±0.18	1.74±0.45	0.64±0.13	0.87±0.17	-0.19±0.17	1.64±0.34	0.65±0.11	1.22±0.30	-0.48±0.30	2.83±0.69	0.40±0.16	1.24±0.31	-0.39±0.30	2.87±0.79	0.36±0.19
P38	0.56±0.06	-0.01±0.06	0.73±0.08	0.87±0.04	0.59±0.07	-0.19±0.07	0.77±0.08	0.87±0.04	0.72±0.09	-0.28±0.09	0.96±0.11	0.80±0.07	1.28±0.14	-0.83±0.14	1.65±0.16	0.65±0.10
PDE2	0.47±0.08	-0.18±0.08	0.65±0.09	0.93±0.02	0.57±0.09	-0.16±0.09	0.77±0.11	0.90±0.03	0.64±0.12	-0.18±0.12	0.93±0.14	0.84±0.06	0.97±0.14	-0.04±0.14	1.25±0.15	0.72±0.08
PTP1B	0.73±0.16	-0.25±0.16	1.33±0.34	0.49±0.21	0.94±0.21	-0.11±0.21	1.72±0.39	0.17±0.25	0.92±0.19	-0.05±0.19	1.62±0.32	0.16±0.22	1.19±0.19	-0.34±0.19	1.79±0.32	0.13±0.20
TYK2	0.16±0.04	0.05±0.04	0.26±0.07	0.99±0.01	0.22±0.05	0.09±0.05	0.32±0.07	0.98±0.01	0.27±0.06	0.12±0.06	0.40±0.07	0.96±0.03	0.68±0.09	-0.01±0.09	0.81±0.11	0.76±0.13
Thrombin	0.30±0.08	0.19±0.08	0.43±0.11	0.91±0.05	0.32±0.08	0.19±0.08	0.45±0.10	0.91±0.05	0.33±0.08	0.20±0.08	0.46±0.10	0.90±0.05	0.33±0.07	0.14±0.07	0.44±0.10	0.91±0.05
All	0.59±0.05	-0.19±0.05	1.18±0.12	0.75±0.04	0.65±0.05	-0.19±0.05	1.25±0.11	0.72±0.04	0.78±0.07	-0.27±0.06	1.65±0.18	0.59±0.06	0.96±0.07	-0.36±0.07	1.78±0.20	0.55±0.07
All except BACE_scaff	0.49±0.04	-0.08±0.04	0.95±0.13	0.81±0.05	0.54±0.04	-0.06±0.04	0.98±0.11	0.80±0.04	0.62±0.05	-0.10±0.05	1.34±0.22	0.67±0.08	0.81±0.06	-0.19±0.06	1.50±0.22	0.62±0.08

Table S3. The subset of ligand-protein systems selected for more extended study in this article.

Protein	Compound pair	vanish	grow
BACE	LCAT-13a – LCAT-13m	18	23
BACE_hunt	L11 – L19	8	12
BACE_p2	L28 – L29	5	6
BACE_scaff	L17a – L27a	21	24
CDK2	L17 – L1h1q	4	4
CMET	LCHEMBL3402744_300_4 – LCHEMBL3402745_200_5	1	4
Galectin	LNHMe – LNMe2	6	9
JNK1	L17124-1 – L18631-1	7	3
MCL1	L26 – L44	17	18
P38	Lp38a_2aa – Lp38a_2bb	10	11
PDE2	L43249674 – L48022468	2	2
PTP1B	L20667 – L23479	24	13
TYK2	L1a – L3b	2	8
Thrombin	Lejm_31 – Lejm_43	2	8

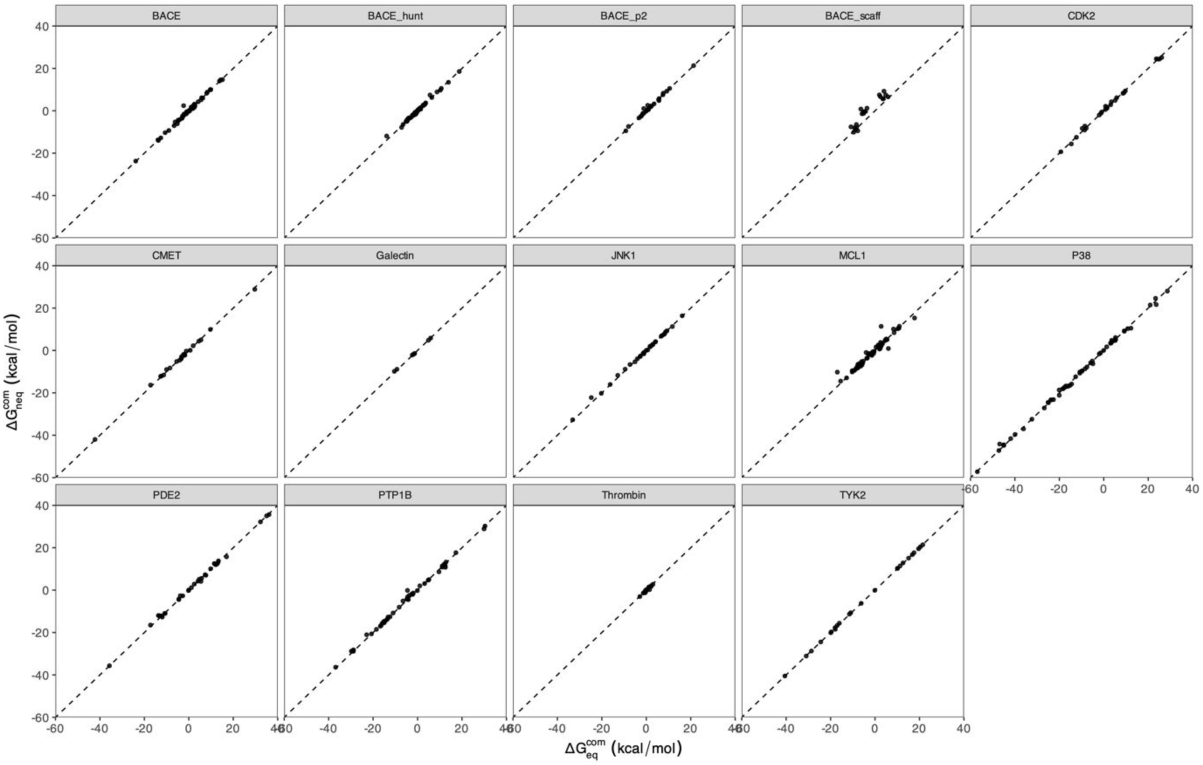

Figure S1. Correlation of free energy changes in the complex leg from the equilibrium and non-equilibrium approaches.

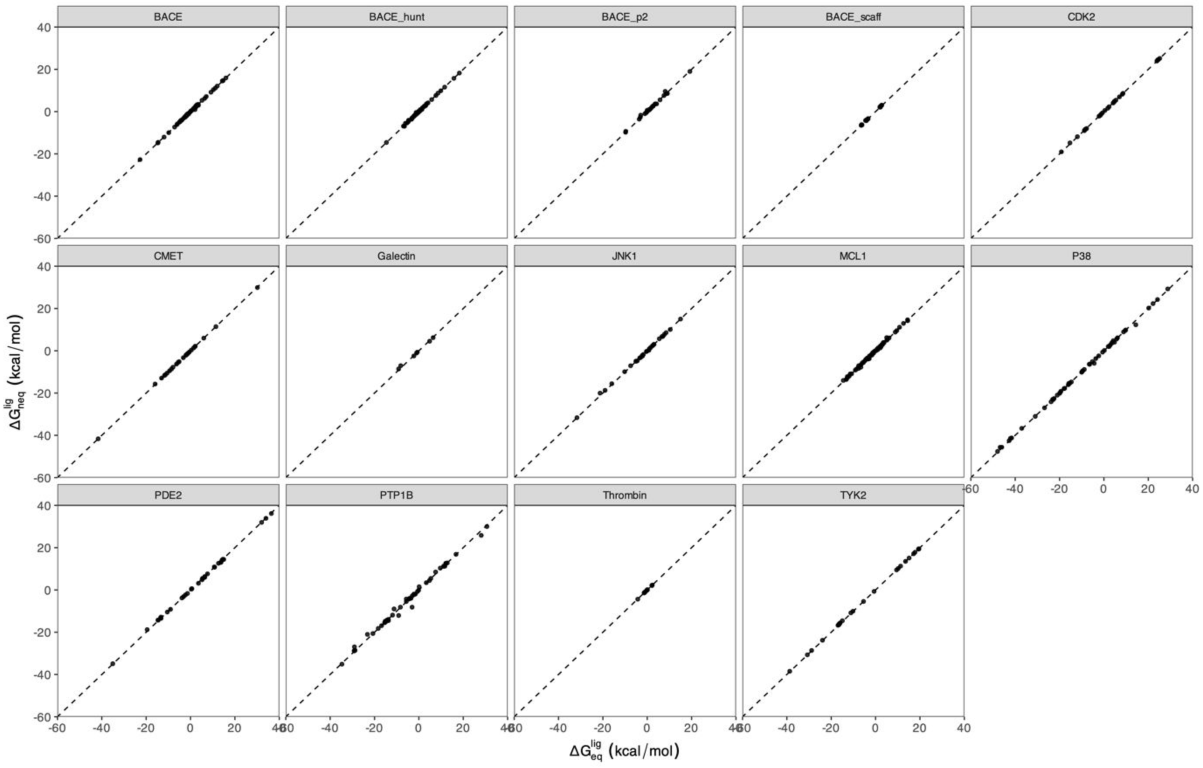

Figure S2. Correlation of free energy changes in the solvent leg from the equilibrium and non-equilibrium approaches.

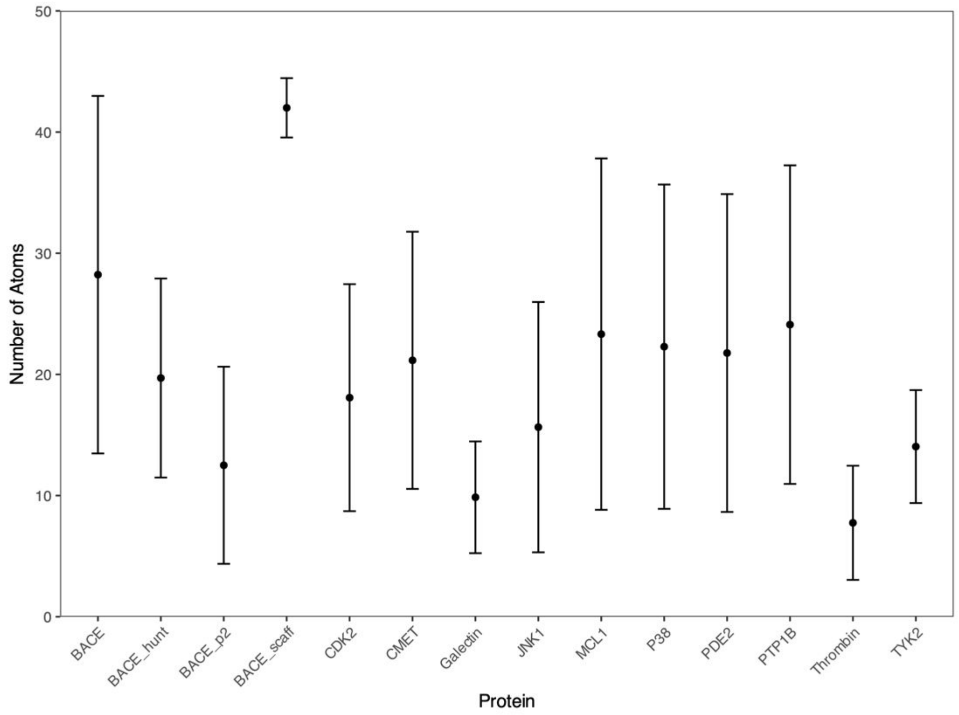

Figure S3. The average number of atoms in the alchemical region and their standard deviations for each protein system. BACE_scaff has many more atoms in the region than other proteins, with an average of 42 atoms comparing with 21 for all other protein systems.

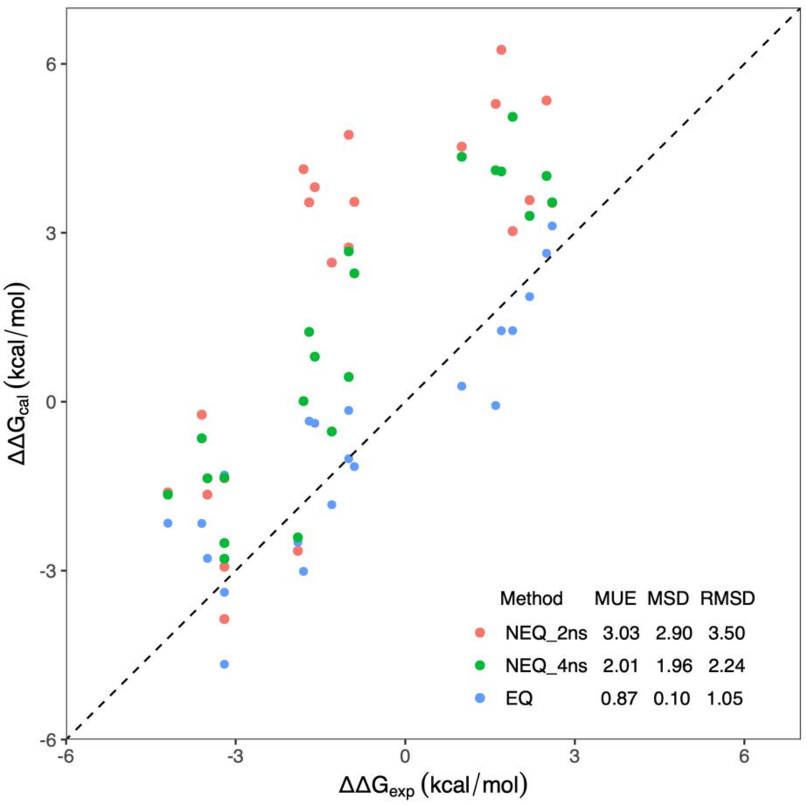

Figure S4. Extension of non-equilibrium simulations from 2 ns to 4 ns for the BACE_scaff system. As compared to the 2-ns long non-equilibrium simulations, significant improvement is observed with the extended 4-ns long runs. This extended duration is, however, still insufficient for the non-equilibrium method to achieve the accuracy obtained with the equilibrium method. All energies are in kcal/mol.

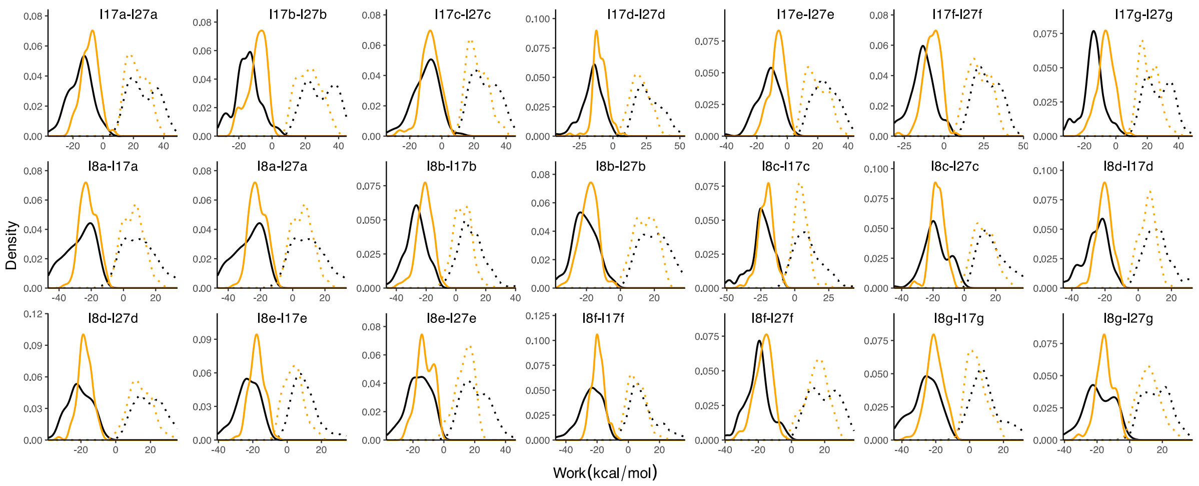

Figure S5. Distributions of the work values from 2 ns (black lines) and 4 ns (orange lines) non-equilibrium transitions for the ligand pairs of BACE_scaff system. The non-equilibrium transitions are performed for both directions, for λ changing from 0 → 1 (dashed lines) and 1 → 0 (solid lines).

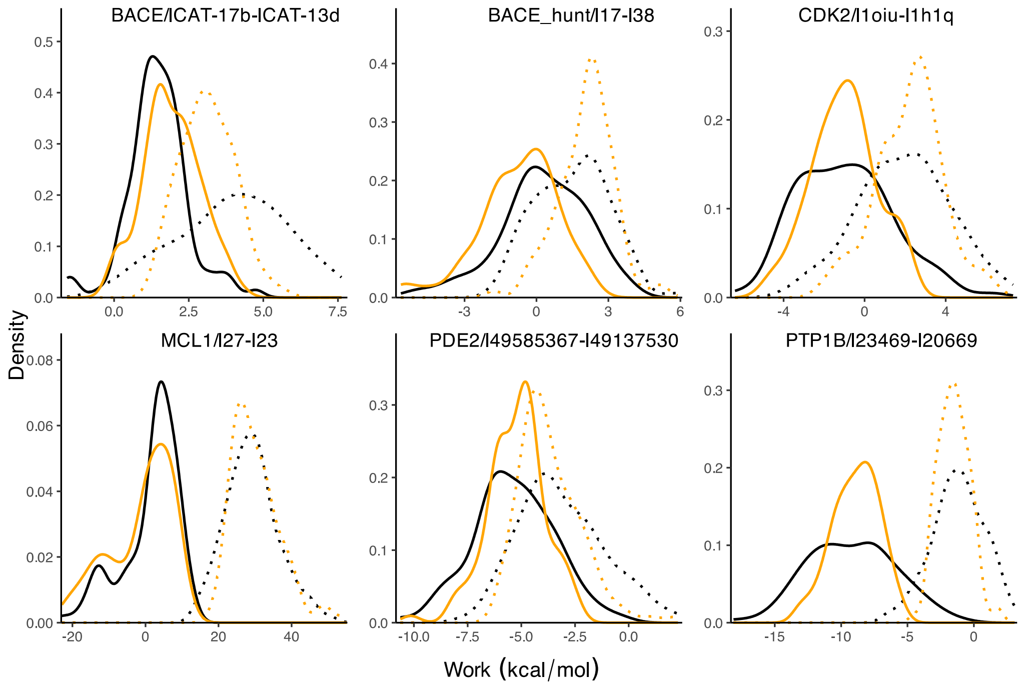

Figure S6. Typical distributions of the work values from 250 ps (black lines) and 2 ns (orange lines) non-equilibrium transitions for some molecular systems. The non-equilibrium transitions are performed for both directions, for λ changing from 0 → 1 (dashed lines) and 1 → 0 (solid lines). Longer simulations generate narrow distributions, with peaks getting closer and tails shrinking for the forward and reverse distributions.

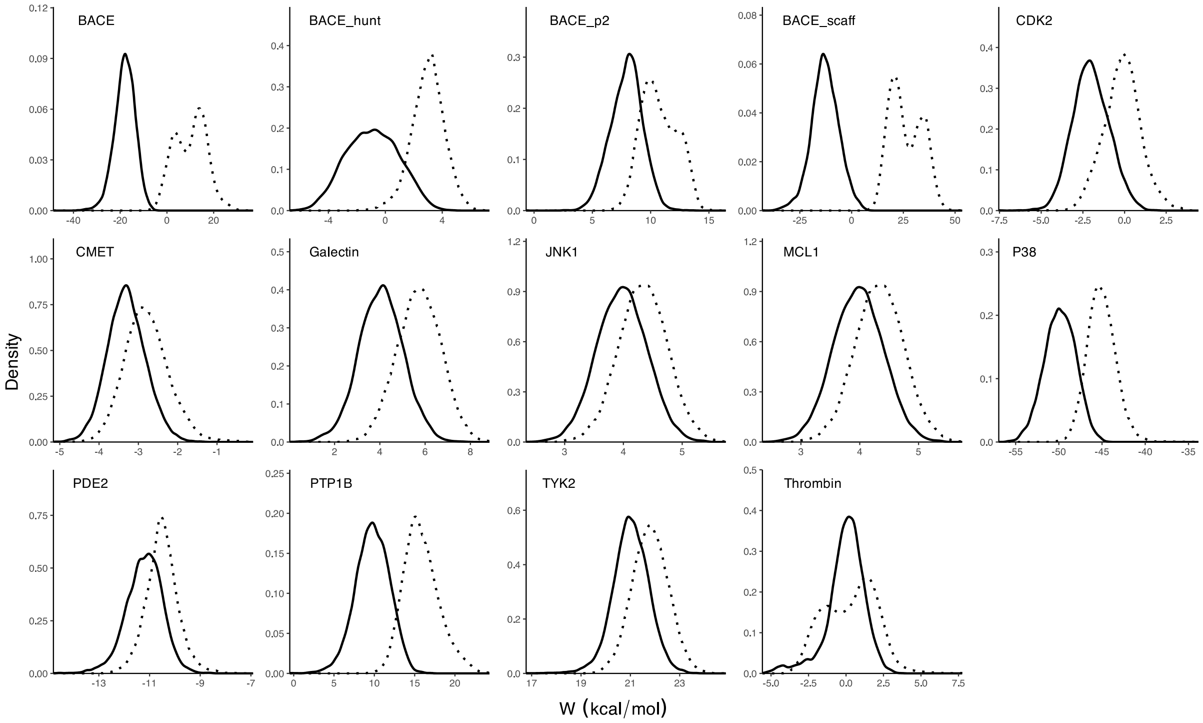

Figure S7. Distributions of the work values from 10,000 individual non-equilibrium transitions for each run. The simulations are started from frames extracted from 20-replica equilibrium runs at the two endpoints. The 2 ns non-equilibrium transitions are performed for both directions, for λ changing from 0 → 1 (dashed lines) and 1 → 0 (solid lines). It should be noted that the scales are different for the non-equilibrium work values W (x-axis).
